# Pediatric Meningeal Diseases: What Radiologists Need to Know

**DOI:** 10.3390/tomography10120143

**Published:** 2024-12-08

**Authors:** Dhrumil Deveshkumar Patel, Laura Z. Fenton, Swastika Lamture, Vinay Kandula

**Affiliations:** 1 Department of Radiology, Nemours Children’s Health, 1600 Rockland Rd., Wilmington, DE 19803, USA; 2 Department of Radiology, Children’s Hospital Colorado, 13123 East 16th Ave., Aurora, CO 80045, USA; 3 Department of Radiology, Seth GS Medical & KEM Hospital, Acharya Donde Marg, Parel, Mumbai 400012, India; swastikalamture@gmail.com

**Keywords:** meningeal enhancement, pediatric, meningitis, drop metastasis, pachymeningeal enhancement

## Abstract

Evaluating altered mental status and suspected meningeal disorders in children often begins with imaging, typically before a lumbar puncture. The challenge is that meningeal enhancement is a common finding across a range of pathologies, making diagnosis complex. This review proposes a categorization of meningeal diseases based on their predominant imaging characteristics. It includes a detailed description of the clinical and imaging features of various conditions that lead to leptomeningeal or pachymeningeal enhancement in children and adolescents. These conditions encompass infectious meningitis (viral, bacterial, tuberculous, algal, and fungal), autoimmune diseases (such as anti-MOG demyelination, neurosarcoidosis, Guillain-Barré syndrome, idiopathic hypertrophic pachymeningitis, and NMDA-related encephalitis), primary and secondary tumors (including diffuse glioneuronal tumor of childhood, primary CNS rhabdomyosarcoma, primary CNS tumoral metastasis, extracranial tumor metastasis, and lymphoma), tumor-like diseases (Langerhans cell histiocytosis and ALK-positive histiocytosis), vascular causes (such as pial angiomatosis, ANCA-related vasculitis, and Moyamoya disease), and other disorders like spontaneous intracranial hypotension and posterior reversible encephalopathy syndrome. Despite the nonspecific nature of imaging findings associated with meningeal lesions, narrowing down the differential diagnoses is crucial, as each condition requires a tailored and specific treatment approach.

## 1. Introduction

Meningeal diseases in pediatric patients encompass a diverse array of pathologies, including infectious, inflammatory, neoplastic, and other etiologies [1]. These conditions often manifest radiologically as abnormal post-contrast enhancement, nodules, basal exudates or diffuse thickening of the meninges [2]. The significant overlap in imaging appearances among these various meningeal diseases, despite their distinct clinical courses and prognoses, presents a diagnostic challenge for radiologists. For example, in a study by Bou et al., exploring the causes of leptomeningeal enhancement (LME), anti-MOG antibody associated demyelination (MOGAD) comprised 5% of the cases; which has significantly different clinical course than the commonly encountered infectious meningitis [3]. Consequently, a systematic imaging approach in tandem with clinical and laboratory data is essential in classifying these for accurate interpretation and optimal patient management.

In this review, we propose an imaging-based classification of pediatric meningeal diseases to facilitate differentiation among various pathologies. This classification system aims to provide a structured framework for radiologists to approach meningeal abnormalities in children. Particular emphasis is placed on parenchymal and other associated imaging features, as these characteristics often play a crucial role in distinguishing between different meningeal pathologies. The objectives of this review are threefold: to present a comprehensive radiological classification of pediatric meningeal diseases, to elucidate the patterns of parenchymal involvement associated with various meningeal pathologies, and to delineate the key radiological and clinical features that aid in differentiating these conditions. It is noteworthy that imaging studies frequently precede lumbar puncture in the emergency department setting, and radiologists are expected to provide a report before cerebrospinal fluid results become available [1,4]. Also, the sensitivity of detecting infectious meningitis in infants, reduces if imaged later in the course of the disease [5]. This underscores the critical importance for radiologists to possess a thorough understanding of the imaging differentials of meningeal diseases, enabling them to provide timely and accurate interpretations that guide clinical decision-making and patient management.

## 2. Imaging Based Classification of Pediatric Meningeal Diseases

**Figure 1:**
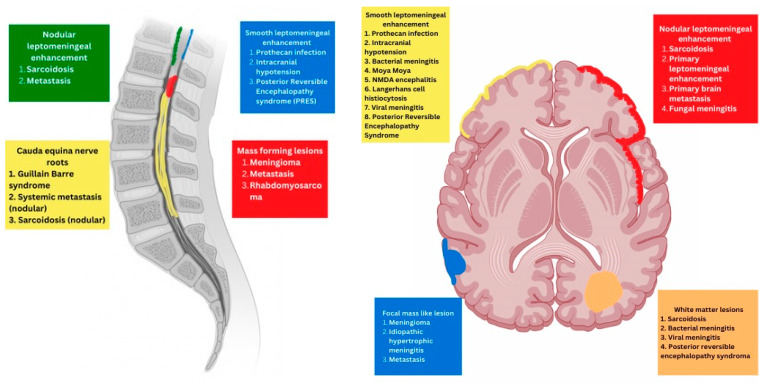
Schematic representation of various radiological appearances.

Radiologically, meningeal pathologies can be broadly divided according to the predominant structures affected (Table 1 and Figure 1). They can be classified as predominantly involving:

1. Meningeal
2. Parenchymal
3. Variable

## 3. Predominant Meningeal Features

### 3.1. Protothecans

**Figure 2:**
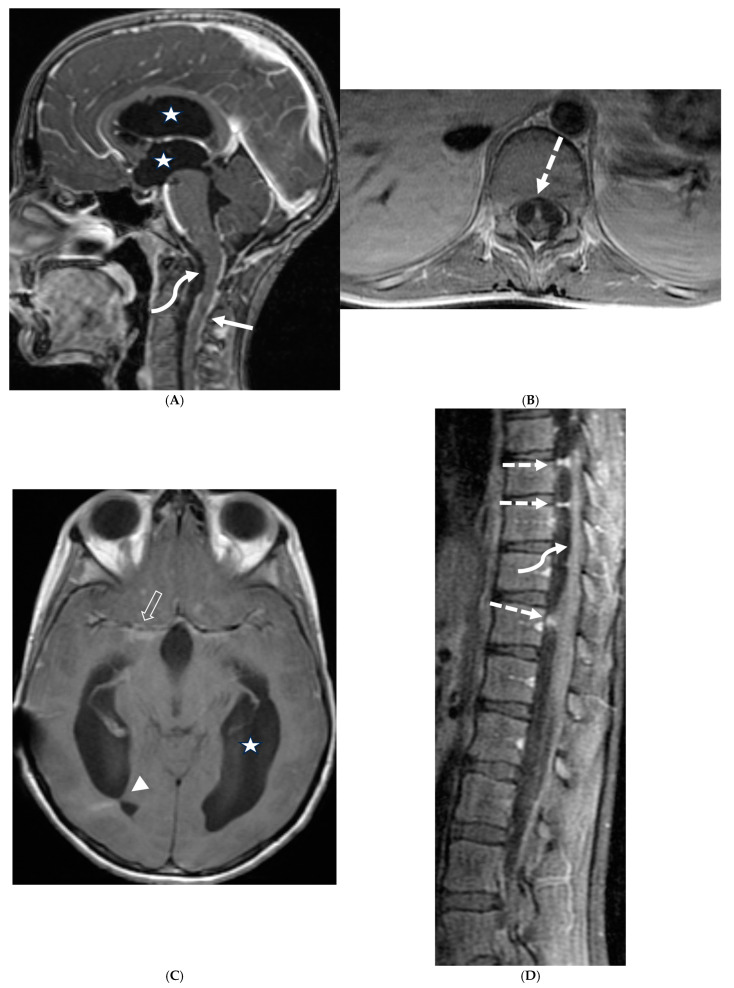
Post contrast sagittal T1 brain (**A**), T1 spine (**B**), axial T1 brain (**C**) and spine (**D**): 17-year-old girl with couple of years of fatigue, shuffling gait, back/lower extremity pain. There is moderate ventriculomegaly (white star). Meningeal enhancement is present around the cervical cord (white arrow). Flattened and deformed brainstem & spinal cord diffusely (curved arrows) and enhancing septae (dashed arrows) within the thecal sac are noted likely from chronic meningitis. Basal cistern enhancement (open arrow) and septae (arrow head) in the lateral ventricles likely reflects sequela of chronic inflammation/infection. Pathology: Prototheca Zopfil.

*Prototheca* species are unicellular algae that are typically known to involve the cutaneous and subcutaneous tissues in humans [6]. Systemic involvement, although rare, can cause meningitis, peritonitis, endocarditis, etc. and is commonly seen in immunodeficient individuals [7]. *Prototheca* spp. infections are usually exogenous and associated with traumatic inoculation from contaminated soil or water. They can also occur from surgery or catheterization, and even insect bites [8]. Exposure leads to chronic granulomatous inflammation with necrosis [6]. Notably, diagnosis of protothecosis may be challenging as it is not easily identified on Hematoxylin and eosin (H&E) or routine fungal stains, and mimics many fungal infections such as *Coccidiodes, Blastomyces*. Imaging findings include diffuse LME along the cortical sulci and spinal cord, with multiple loculations given the chronic inflammatory response. These loculations characteristically cause mass effect leading to a flattened and deformed spinal cord (Figure 2) [9].

### 3.2. Neurosarcoidosis

Sarcoidosis is a systemic inflammatory disorder characterized by non-caseating granuloma formation [10,11,12]. Neurosarcoidosis (NS) is uncommon, detected on imaging studies in 15% of the patients, out of which only one-third of them present with clinical symptoms [10,13]. Granulomas, a hallmark of sarcoidosis, can infiltrate cerebral parenchyma, brain vasculature, and cranial nerves [14,15]. Parenchymal involvement leads to motor or sensory deficits, whereas predominant meningeal and subarachnoid involvement leads to cranial nerve deficiencies and vision changes [11]. Contrast-enhanced MRI of the brain and/or the spine is currently considered the standard of care for initial work-up and follow-up in NS [16].

**Figure 3:**
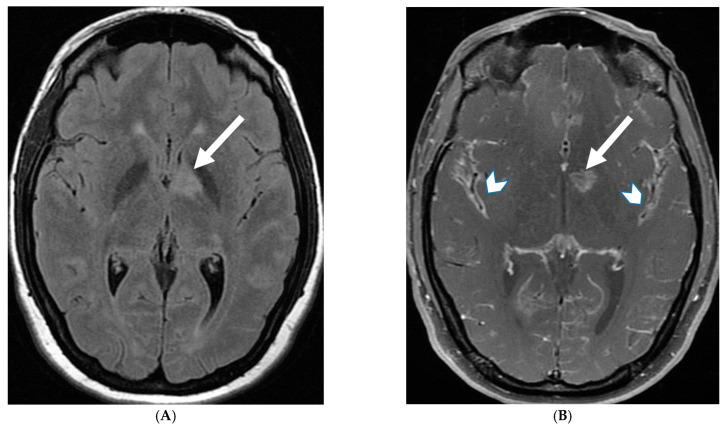

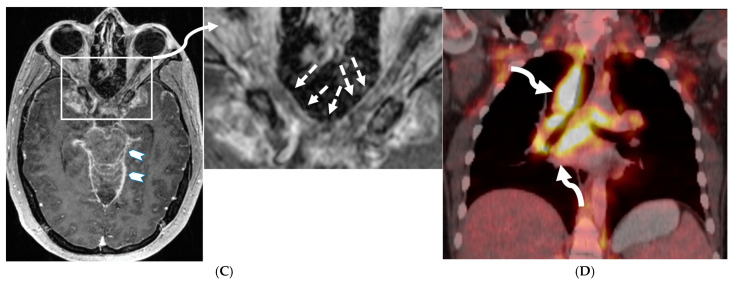
18-year-old presented with headache, persistent vomiting and weight loss. History of sarcoidosis diagnosed 2 years ago. Axial Fluid Attenuated Inversion Recovery (FLAIR) (**A**), Axial T1 post contrast (**B**), Axial T1 inversion recovery post contrast (**C**) and Coronal Positron Emission Tomograpy (PET) scan (**D**): There is a heterogeneously enhancing ill-defined area of T2/FLAIR hyperintensity involving the medial aspect of the left globus pallidus (arrows), anterior aspect of the left thalamus and left hypothalamic region. Diffuse enhancement of the basal meninges, tentorium, throughout perisylvian sulci (arrow heads), along the infundibulum, and posteriorly at the craniocervical junction. There is also enhancement along optic nerve sheath (dashed arrows). Features are highly consistent with extensive neurosarcoidosis given the previous history of thoracic sarcoid. PET scan from 2 years earlier demonstrating avid uptake of radiotracer (curved arrows). Radiologically, the differential diagnosis includes tuberculosis and metastatic process. Patient made complete recovery after treatment for sarcoid.

Nodular or diffuse LME, primarily involving the basal meninges, is the most typical finding. It can further spread into the parenchyma via the perivascular spaces [17]. Most cases show focal involvement and are hypointense on T2WI with variable post contrast enhancement [18,19]. Nonenhancing white matter lesions (NEWM), although common, have been shown to have no symptomatic correlation [18]. Optic and facial nerves are frequently involved (Figure 3). The diagnosis of optic neuritis is crucial and regarded as an emergency due to its unfavorable prognosis if not promptly treated [20]. Occasionally [18,21,22], small vessel ischemia related cerebrovascular events occur which manifest clinically with progressive encephalopathy rather than a distinct large vessel stroke [13]. Other rare but important findings include spinal cord and hypophyseal involvement [23]. The mainstay of treatment for CNS sarcoidosis involves corticosteroids to suppress inflammation.

### 3.3. Guillain Barre Syndrome

Guillain-Barré syndrome (GBS) is a rapidly progressive autoimmune disorder affecting the peripheral nervous system usually in response to a prior respiratory or gastrointestinal infection [24,25,26,27,28]. The hallmark presentation of GBS is progressive ascending weakness typically beginning in the legs and spreading to the arms. Areflexia, autonomic dysfunction, and respiratory failure can also occur [29]. Diagnosis is primarily based on clinical presentation, supported by cerebrospinal fluid (CSF) analysis and electrophysiological studies [30,31]. Magnetic resonance Imaging (MRI) is indicated in equivocal cases where excluding other diagnosis is critical and would alter management.

**Figure 4:**
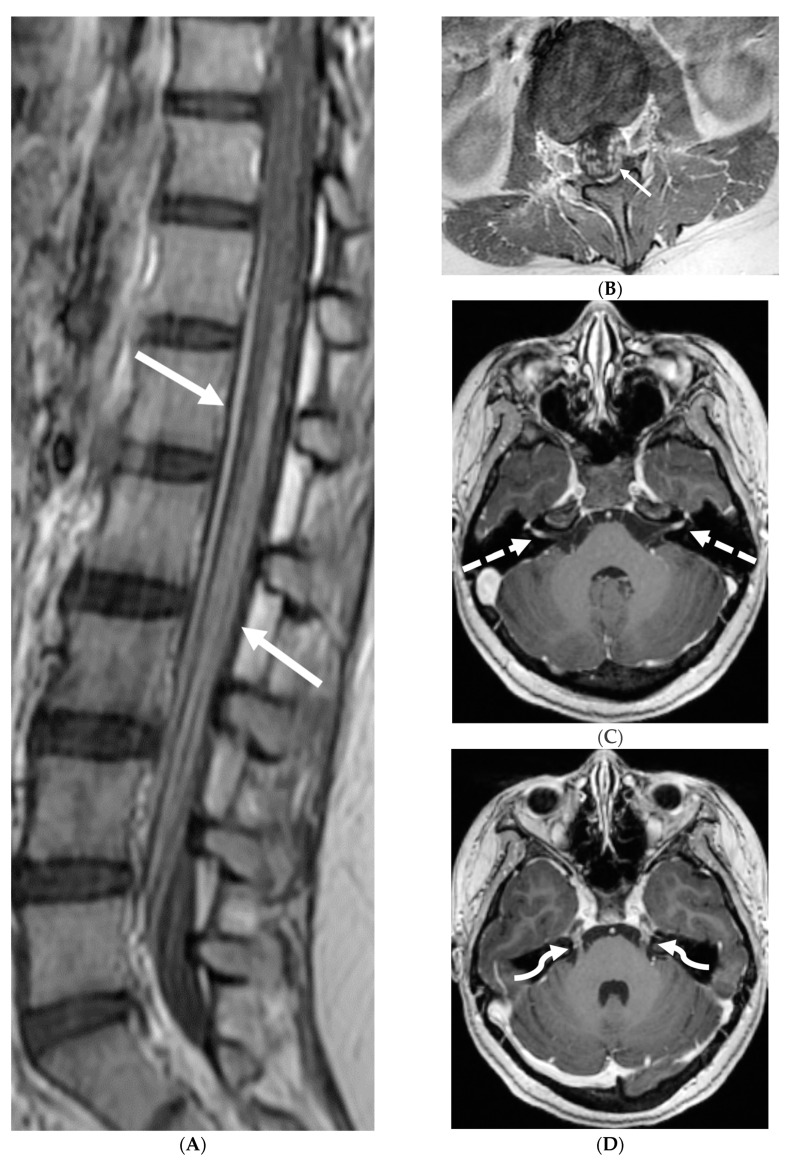
Post contrast sagittal T1 (**A**) and axial T1 (**B**) of the lumbar spine, post contrast axial T1 of the brain (**C**,**D**): 14-year-old girl with numbness/tingling, paresthesia and bilateral lower extremity weakness. Patient also has bilateral facial weakness. There is diffuse mild thickening of the cauda equina nerve fibers with enhancement (arrows). Additionally, exiting nerve roots of the cervical and thoracic region also show enhancement. Enhancement of bilateral facial (dashed arrows) and trigeminal nerves is also visualized (curved arrows). Features are in keeping with Guillain-Barre syndrome (acute inflammatory demyelinating polyneuropathy). With involvement of facial and trigeminal nerves, Miller Fisher variant should be considered.

The most characteristic MRI findings in GBS are smooth contrast enhancement of the spinal nerve roots with variable thickening, particularly in the cauda equina region (Figure 4). Selective or prominent anterior nerve root enhancement favors the diagnosis of GBS [32,33]. A higher incidence of cranial nerve abnormalities, particularly the optic nerve, is seen in children with the GBS variant, Miller Fisher syndrome (MFS) [34,35]. Ultrasound imaging of peripheral nerves offers a promising new tool for early GBS diagnosis by detecting enlarged cervical nerve roots early in the disease course [36,37].

### 3.4. Idiopathic Hypertrophic Meningitis (IHP)

Hypertrophic pachymeningitis (HP) is a rare disorder characterized by localized or diffuse thickening of the dura mater without an attributable cause [38,39,40]. Recent studies suggest a possible link between IHP and IgG4-related disease (IgG4-RD) [40,41,42].

The clinical manifestations of IHP vary depending on the location of the thickened dura and resulting nerve compression. Vertebral canal involvement may cause radiculopathy, limb weakness, and sphincter dysfunction [43]. Anterior cranial fossa involvement may present with retro orbital pain, decreased visual acuity, and eye movement disturbance (due to involvement of cavernous sinus or superior orbital fissure). Posterior fossa involvement may cause dysfunction of cranial nerves VI to XII (most common cranial nerve involved is VIII), and cerebellar ataxia [40].

Cross sectional imaging is marked by a thickened hyperdense dura on non-enhanced Computed Tomography (CT), typically along the tentorium, falx, and prepontine brainstem [44]. MRI typically shows relatively hypointense signal on both T1-weighted and T2-weighted images. Contrast-enhanced T1-weighted MR images characteristically reveal marked homogenous or peripheral dural enhancement [44,45].

While meningioma en plaque and tuberculoma en plaque can also thicken the dura mater, their involvement is typically localized rather than diffuse. Additional parenchymal abnormalities (except brain edema) are absent in IHP [44]. Additionally, these conditions usually cause symptoms from mass effect, and not due to entrapment of nerves and blood vessels [46].

### 3.5. Meningioma

Pediatric meningiomas account for less than 5% of childhood brain tumors, with a higher incidence in the second decade of life [45]. They are associated with neurofibromatosis (NF) types 1 and 2, as well as prior radiation therapy [46]. While it was previously thought that pediatric meningiomas had a higher likelihood of being atypical, this is now a topic of debate [47]. Clinical symptoms are non-specific and vary depending on the tumor’s location. Although convexity and parasagittal locations are more common, meningiomas can also be found in atypical locations such as the skull base and ventricles [48]. Meningiomas at the craniocervical junction are typically associated with NF-2 [49].

**Figure 5:**
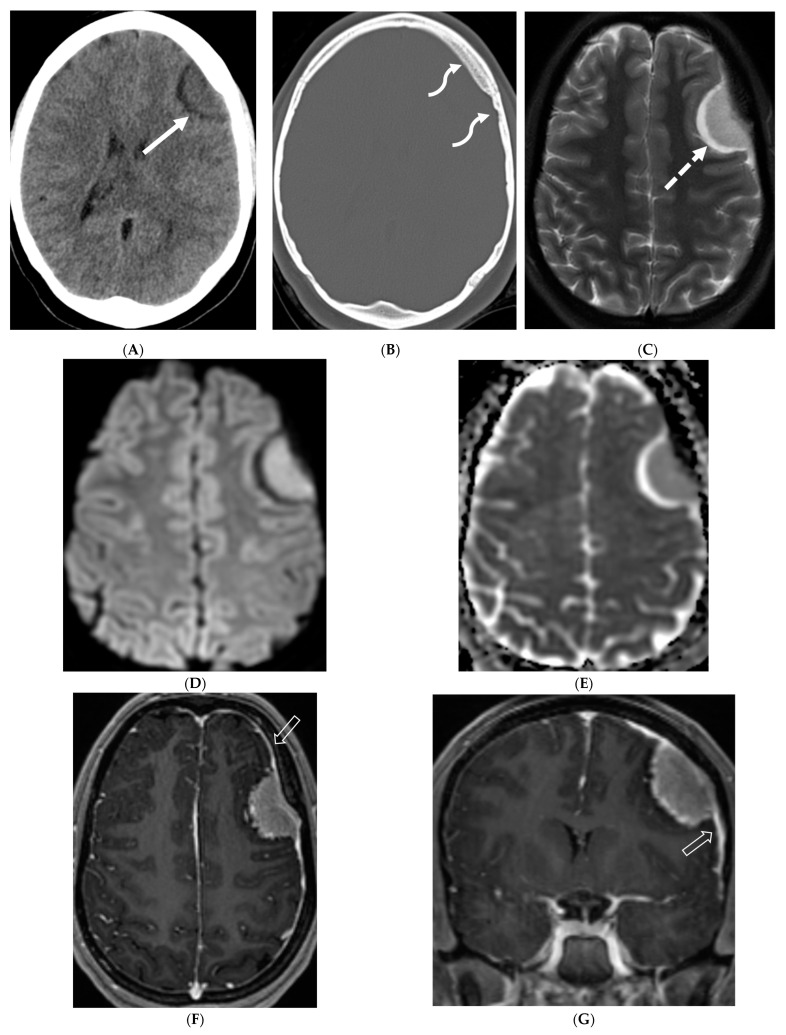
Axial CT (**A**,**B**), axial T2 fat saturated (FS) (**C**), Axial Diffusion Weighted Imaging (DWI) & Apparent Diffusion Coefficient (ADC) (**D**,**E**), axial and coronal post contrast (**F**,**G**): 15-year-old girl with nausea and headaches. CT shows iso-dense dural-based mass in the left anterior cranial fossa (arrow). Adjacent bone is hyperostotic and has irregular cortex (curved arrow). The lesion is isointense with cortex, which is buckled inwards from the mass. A hyperintense rim surrounds the mass representing CSF cleft (dashed arrow). No significant restricted diffusion is noted. The mass enhances intensely and uniformly. A dural tail (open arrow) of benign, nonneoplastic reactive thickening is present adjacent to the left frontal mass, characteristic of classic “typical” WHO grade 1 meningioma.

Several imaging features of pediatric meningiomas are similar to those seen in adults: the majority are of the meningothelial type, displaying a hyperdense core on non-enhanced CT (NECT), isointensity to gray matter on T1- and T2-weighted MRI sequences (Figure 5), and moderate post-contrast enhancement. The detection of one meningioma should prompt a thorough search for additional tumors, as one-third of cases are known to be multiple, indicating potential syndromic or radiation-induced associations [49]. A dural tail is less commonly observed, and its absence does not exclude the presence of a meningioma (Figure 5) [49]. Cystic components are more frequently seen in pediatric meningiomas [49]. Intratumoral calcifications and hyperostosis are present in approximately half of the cases [50]. Imaging differentials to consider include dural LCH or Ewing’s sarcoma [51].

### 3.6. Glioneuronal Tumor

Diffuse leptomeningeal glioneuronal tumor (DL-GNT) is a recently classified brain tumor (WHO 2016) previously known by various terms such as disseminated oligodendroglial-like leptomeningeal tumor, dysembryoplastic neuroepithelial tumor-like neoplasm and meningeal gliomatosis [52]. It is also associated with precancerous conditions such as KIAA1549-BRAF gene fusion, 1p deletion or 1p/19q co-deletion and Haberland syndrome [53,54].

**Figure 6:**
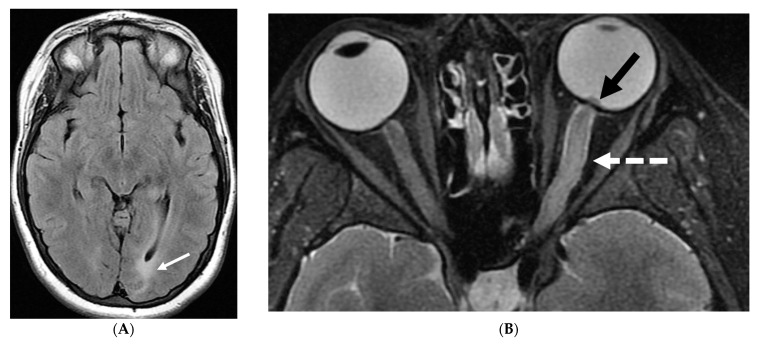

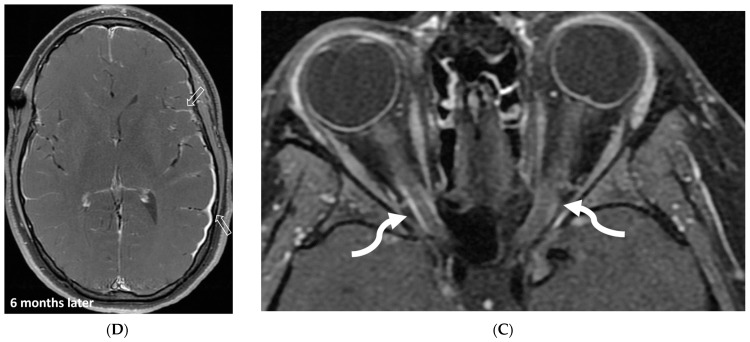
Axial FLAIR (**A**), Axial T2 orbits (**B**), Axial T1 orbits post contrast (**C**) and axial T1 post contrast (**D**): 13-year-old female with headache and blurred vision. Abnormal FLAIR hyperintensity involving the left parieto-occipital periventricular white matter (arrow), and bilateral cerebellar hemispheres. Bilateral papilledema (black arrow) and edematous left optic nerve (dashed arrow). Peripheral optic nerves/optic sheath enhancement in the posterior aspect (curved arrows). Demyelination, infectious and metastatic processes were considered. MRI brain 6 months later with persistent symptoms demonstrates patchy and asymmetric pachymeningeal and leptomeningeal enhancement (open arrows). Pathology: Diffuse Leptomeningeal Glioneuronal Tumor.

Although a low-grade neoplasm, leptomeningeal spread is the norm [55]. DL-GNT is characterized by diffuse leptomeningeal thickening, often with basal predominant nodular enhancement [56]. There is invariable involvement of the leptomeninges along the spinal cord in linear fashion [57]. Distinctively, numerous small T2-hyperintense parenchymal cysts are present as a result of fibrosis and obstruction in the subarachnoid space; typically in the inferior frontal and medial temporal lobes [53]. These cysts show incomplete signal suppression on T1 and FLAIR images, possibly reflecting their mucoid nature [53,57]. Engulfment of peripheral nerve roots and invasion of choroid plexus may be seen (Figure 6) [55]. The diagnosis of DL-GNT be pursued with characteristic imaging findings with infectious etiology been ruled out [56].

### 3.7. Primary Leptomeningeal Rhabdomyosarcoma

Rhabdomyosarcoma, the most common childhood soft tissue sarcoma, is commonly seen in the head and neck, genitourinary tract and extremity [58]. Primary meningeal rhabdomyosarcoma is extremely rare [59,60]. It is hypothesized that the origin of this rare variant is cerebral parenchyma with secondary leptomeningeal spread [61].

Diffuse LME with areas of leptomeningeal thickening and nodularity would be the prominent imaging finding which may cause hydrocephalus. These findings mimic more common entities such as infection (e.g., tuberculosis) or inflammation (e.g., neurosarcoidosis).

**Figure 7:**
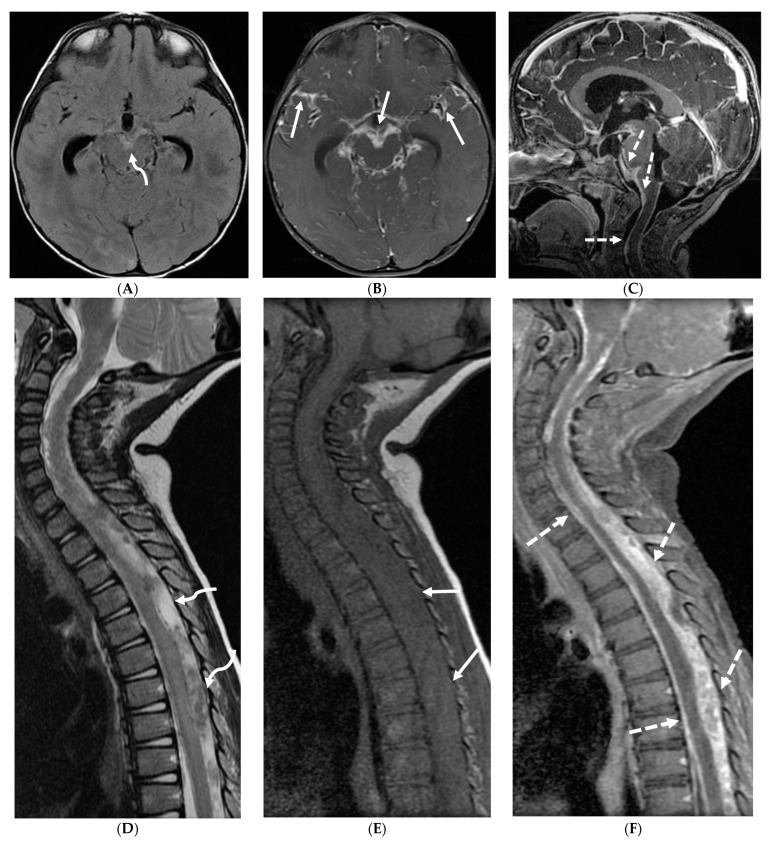
3-year-old boy with 2 weeks history of headache and vomiting. Axial FLAIR (**A**), post contrast axial T1 FS (**B**) and sagittal 3D inversion recovery (**C**) images demonstrate FLAIR hyperintensity in the interpedencular cistern (curved arrow) and mild hydrocephalus. Extensive meningeal enhancement most prominent at the skull base, basal cisterns, and Sylvain fissures (arrows), but extending throughout the brain. There is meningeal enhancement, with coating of the brainstem extends inferiorly along the cervical spinal cord (dashed arrows). Sagittal T2 (**D**), sagittal T1 (**E**) and fat saturated T1 post (**F**) images show extensive leptomeningeal with predominantly solid and some cystic nodules (curved arrows) on T2 sequence and isointense on T1 (arrows). Lesions predominantly involve the posterior spinal canal, causing mass effect and anterior displacement of the spinal cord. The solid nodules show enhancement after contrast injection and extensive uniform diffuse LME around the cord (dashed arrows). Pathology: Primary Meningeal Rhabdomyosarcoma.

Marked focal nodularity and mass effect causing a deformed contour on the spinal cord favor a neoplastic process (Figure 7) [62]. Accurate staging is crucial as the presence of leptomeningeal or multifocal disease have implications on radiotherapy fields and total dose. Additionally, PET-CT scan assists in evaluating for an extracranial primary site [63].

### 3.8. Intracranial Hypotension (IH)

Intracranial hypotension in children is frequently secondary to iatrogenic causes including lumbar punctures, craniospinal surgeries and ventricular shunt drain pressure changes [64]. Spontaneous causes are commonly connective tissue disorders such as Marfans and Ehler Danlos syndromes. Dural tears and meningeal diverticula have been demonstrated in these cases [65]. Beyond headaches, IH can manifest with nausea, vomiting, light sensitivity (photophobia), and stiff neck [66].

MRI features of IH can be explained by the Monroe-Kellie doctrine, which states that the intracerebral volume including blood, CSF and brain parenchyma remain the same. Thus, a decrease in CSF volume promotes dilatation and rounding of the venous sinuses, subdural fluid collections along with dural (pachymeningeal) enhancement which occurs due to vascular engorgement and transudation of fluid into it [67]. Hyperemia of the pituitary gland occurs which may mimic hyperplasia or pituitary tumor. Brainstem slumping or downward displacement of the brainstem, defined as red nuclei below the tentorium and low lying third ventricle below the sella, are highly specific indicators of IH, observed in half the cases. A pontomesencephalic angle of less than 50 degrees and mamillo pontine distance of less than 5.5 mm are sensitive and specific parameters to suggest IH [66].

**Figure 8:**
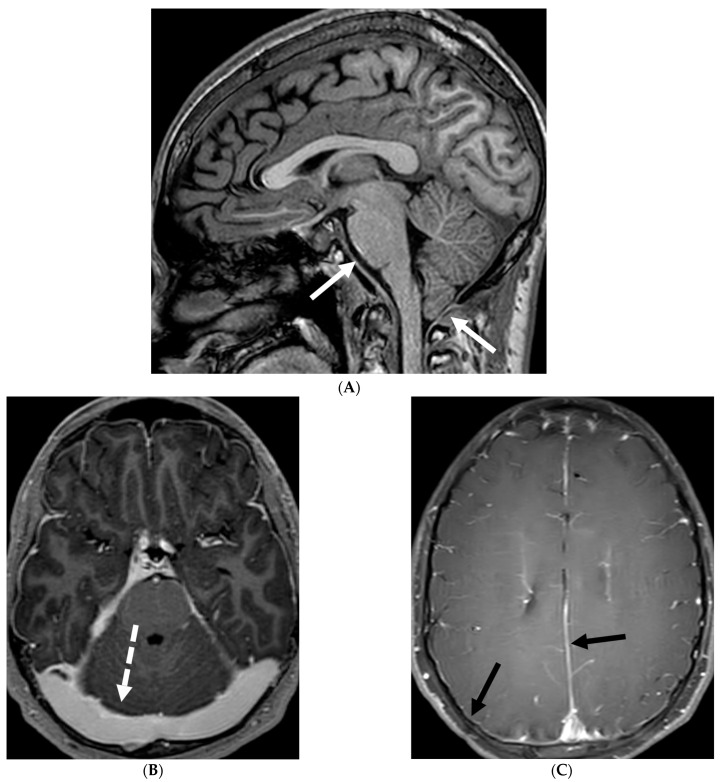

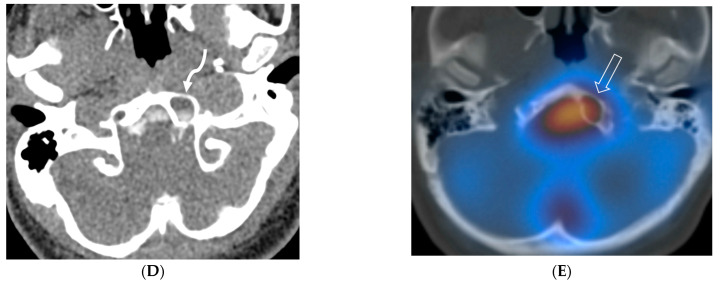
Sagittal T1 (**A**), post axial T1 (**B**,**C**), axial CT myelogram (**D**) and technetium 99 m DTPA SPECT-CT (**E**): 16-year-old with Gorham’s disease. There is cerebellar tonsillar herniation and decrease in prepontine cisterns (white arrows). Significant increase in the size of the venous sinuses (dashed arrow). Diffuse pachymeningeal enhancement is seen (black arrows). Cystic-appearing foci at the skull base are in keeping with lymphangiomatosis with contrast pooling into the lytic lesion (curved arrow). Abnormal radiotracer extravasation in the left clival region correlating with lytic lesion (open arrow). Features are in keeping with intracranial hypotension secondary to CSF leak.

**Figure 9:**
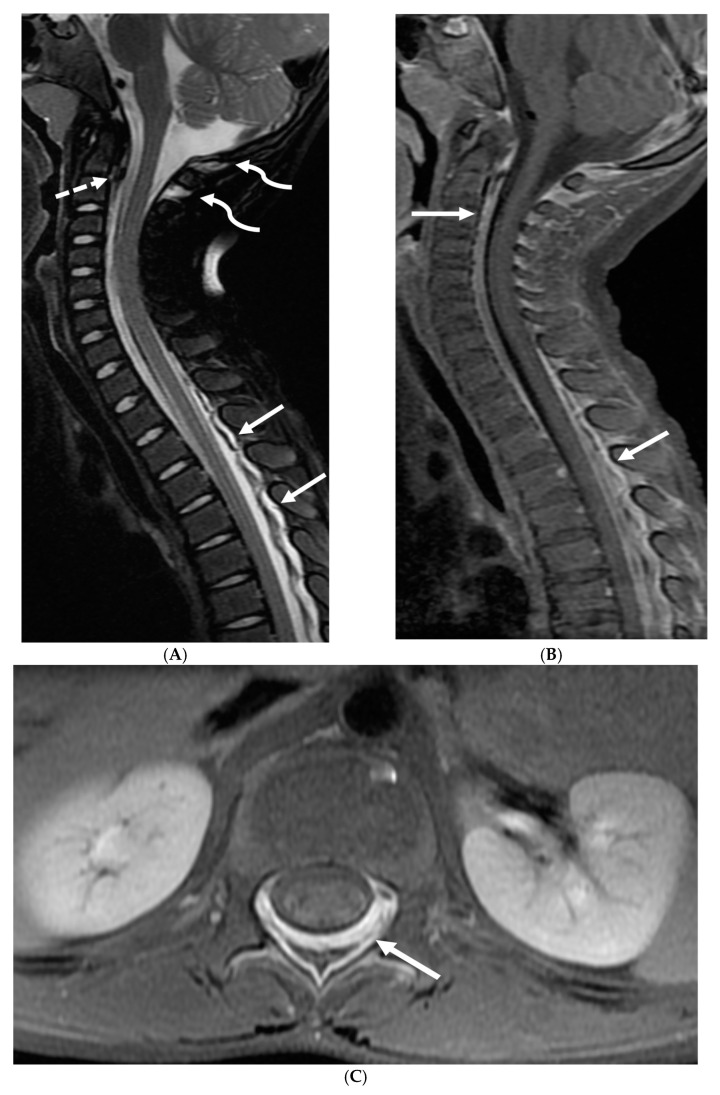
Sagittal T2 FS (**A**), sagittal (**B**) and axial (**C**) T1 post contrast: 3-year-old with neck pain post LP. There is diffuse epidural thickening, with increased T2 signal and enhancement, throughout the cervical, thoracic and lumbar spine (white arrows). Several prominent flow voids are seen within the anterior epidural thickening in the upper cervical region (dashed arrow). There is also increased high T2 signal between the occiput and posterior arch of C1, and between the posterior arch of C1 and spinous process of C2 (curved arrows) in keeping with “C1–C2 sign”. Findings are related to intracranial hypotension post lumbar puncture.

With regard to spine imaging, in addition to the intracranial features of dural enhancement, venous engorgement and subdural collection, unique findings include meningeal diverticula, dural ectasia and C1–C2 sign (Figure 8 and Figure 9). Additionally, a CT myelogram may identify the precise location of the CSF leak which can be sealed off with a blood patch [65,68].

### 3.9. Alk-Positive Histiocytosis

**Figure 10:**
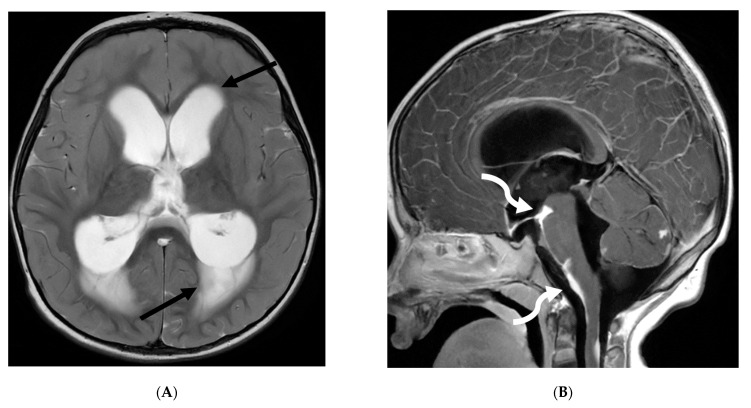

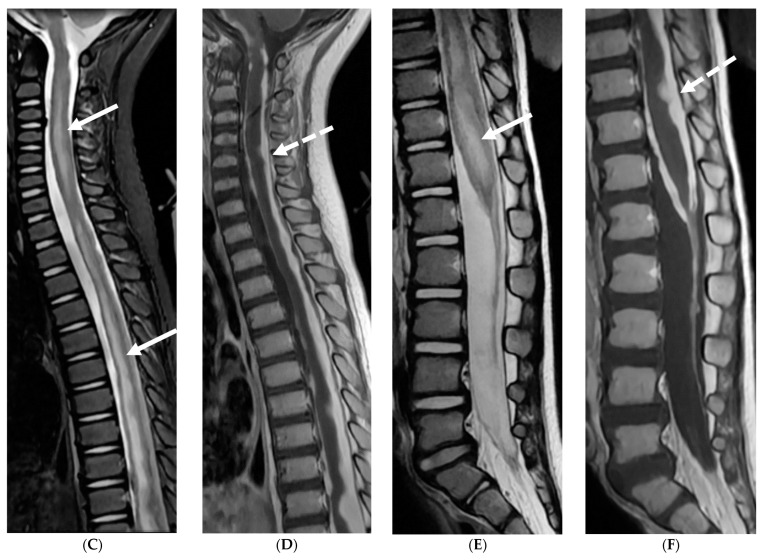
Axial T2 (**A**), sagittal T1 post contrast (**B**), Sagittal T2 (**C**,**E**) and sagittal T1 post contrast (**D**,**F**): 20-month-old boy with 2 months of losing developmental milestones and 1 month of emesis, fatigue and dehydration. Ventriculomegaly with transependymal fluid is noted (black arrows). There is posterior fossa leptomeningeal nodular enhancement extending into the upper cervical spine (curved white arrows). Extensive nodular enhancement along spinal cord (dashed white arrows) with cord edema demonstrated in the entire cord (white arrows).

**Figure 11:**
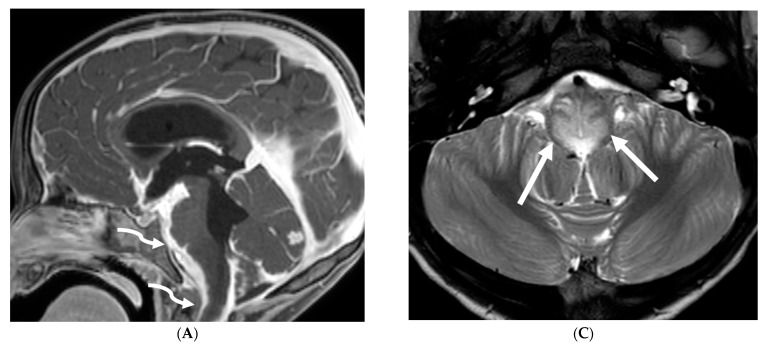

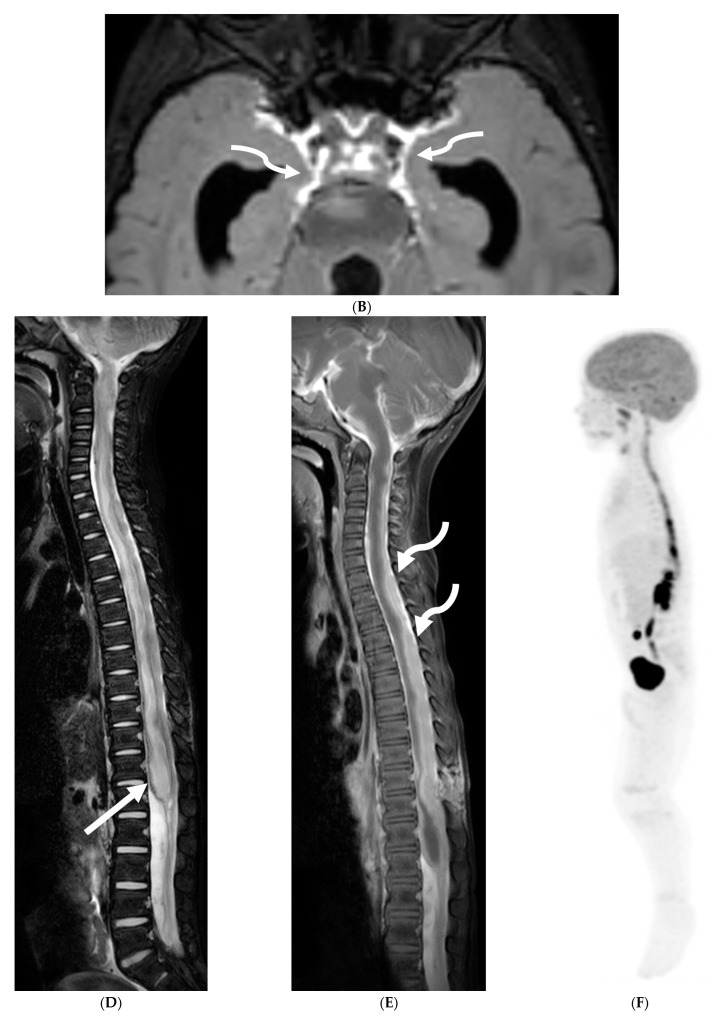
3 weeks follow up: Post contrast sag T1 (**A**) and axial FLAIR (**B**), Axial T2 (**C**), sagittal T2 (**D**), Post contrast sagittal T1 (**E**) and PET/CT (**F**): Leptomeningeal nodular enhancement along posterior fossa, suprasellar and spinal cord (curved arrows) has significantly increased. There is also new/increased signal abnormality in the brain stem and cord (white arrows). Hypermetabolic spine disease is demonstrated on PET/CT. No osseous involvement is identified on the PET scan. Pathology: Diffuse CNS ALK (Anaplastic Lymphoma Kinase)-Positive Histiocytosis. Bone marrow biopsies, US abdomen and skeletal survey negative for extracranial/extraspinal disseminated disease.

ALK-positive histiocytosis (APH) is a rare, non-Langerhans cell histiocytosis that can involve the nervous system, including the meninges. While the disease is often seen in infants and young children, it can occur at any age [68]. Neurologic involvement usually presents as seizures, ataxia, headaches, and vomiting [69]. Imaging findings on CT include iso-dense or slightly hyperdense nodules/masses [70]. On MRI, lesions demonstrate isointense or slightly hypointense signals on T1-weighted, isointense or hypointense signals on T2-weighted, with moderate homogeneous focal or smooth ring enhancement [71]. Meningeal enhancement (Figure 10F) can appear segmental and be particularly prominent along the cauda equina [71]. Restricted diffusion is also commonly seen (Figure 10 and Figure 11) [69].

Differentiating APH from other meningeal diseases can be challenging. The most important imaging differential is meningioma. Meningiomas are typically well-circumscribed, dural-based lesions that enhance intensely and homogeneously. Meningiomas often demonstrate a “dural tail”—a thin, linear enhancement extending from the main tumor mass along the dura mater [71]. Another important differential is juvenile xanthogranuloma (JXG), which can also involve the meninges. JXG lesions are typically well-defined, enhancing nodules that are often located near the ventricles or meninges [71]. Ultimately, a definitive diagnosis of APH requires histopathologic and molecular testing, which demonstrates the presence of ALK rearrangement, typically a KIF5B-ALK fusion [72].

## 4. Variable Meningeal and Parenchymal Features

### 4.1. Bacterial Meningitis

Hematogenous dissemination is the primary cause of bacterial spread to the CNS. The most common infective organisms differ according to age with Group B Streptococcus and Escherichia coli commonly affecting neonates and Streptococcus pneumoniae, Neisseria meningitidis, Haemophilus influenzae (decreasing due to vaccination) occurring in infants and children. Post contrast T1 and FLAIR sequences are the primary ones to demonstrate LME [73]. However, multiple associated parenchymal abnormalities occur in childhood, particularly in neonates due to different causative organisms.

Group B streptococci causes ischemic infarcts along vascular territories in an asymmetric fashion in neonates. Non diffusion restricting extra-axial collections can also occur which tend to resolve on follow up imaging without any sequelae. An important imaging differential in this age group is of hypoxic ischemic encephalopathy (HIE), often with symmetric diffusion restriction in metabolically active regions [74].

**Figure 12:**
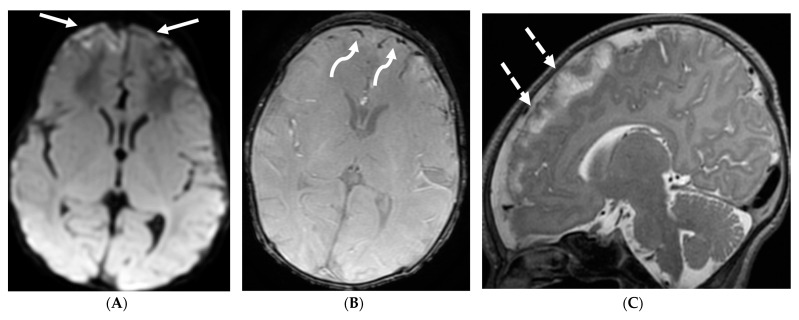

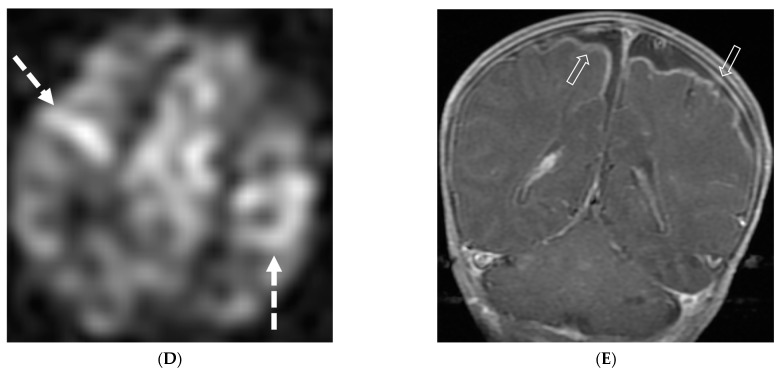
Axial DWI (**A**), axial SWI (**B**), sagittal T2 (**C**), axial ASL (**D**) and coronal T1 post contrast (**E**): 11-day-old female presented with seizures and lethargic. Restricted diffusion is noted in the sulci along the bilateral frontal convexities, concerning for meningitis (arrows). Curvilinear susceptibility in the extra-axial spaces of bilateral frontal convexities, consistent with thrombosed cortical veins (curved arrows). Cortical T2 hyperintensity is seen in the bilateral frontal and parietal lobes with corresponding hyperperfusion in keeping with extensive cerebritis (dashed arrows). Diffuse leptomeningeal and pachymeningeal enhancement is seen (open arrows). Overall features represent meningitis and cerebritis. Cerebrospinal fluid analysis: Group B streptococcus.

*E. coli* is another common neonatal and infantile cause of bacterial meningitis. There is characteristic diffusion restriction in the extra-axial collections along with ventriculomegaly and/or ventriculitis [74]. To note, extra-axial empyema is also common with *S. pneumoniae*, but occur in an older age group. *Serratia marcescens* and *Citrobacter* are two uncommon pathogens causing meningitis in neonates with fulminant disease course. They lead to large parenchymal abscesses with striated appearance on T2W images and foci of susceptibility. *N. meningitidies* causes typical gyriform cortical enhancement consistent with cerebritis predominantly involving the occipital lobes (Figure 12) [74].

### 4.2. Tuberculosis

Tuberculous meningitis (TBM) is a severe form of extrapulmonary tuberculosis, associated with high morbidity and mortality rates in those under 5 years old. Case fatality rate reaches up to 20% and only one-third of the patients having no long term neurological sequelae [75]. In developed countries, while the overall incidence of tuberculosis has declined, TBM remains a concern in pediatric populations, particularly among high-risk groups such as immunocompromised children and those from TB-endemic regions. Clinical presentation in children can be subtle and nonspecific, especially in younger age groups. Initial symptoms may include low-grade fever, irritability, poor feeding, and vomiting, which can progress to more severe manifestations such as altered mental status, focal neurological deficits, and seizures.

**Figure 13:**
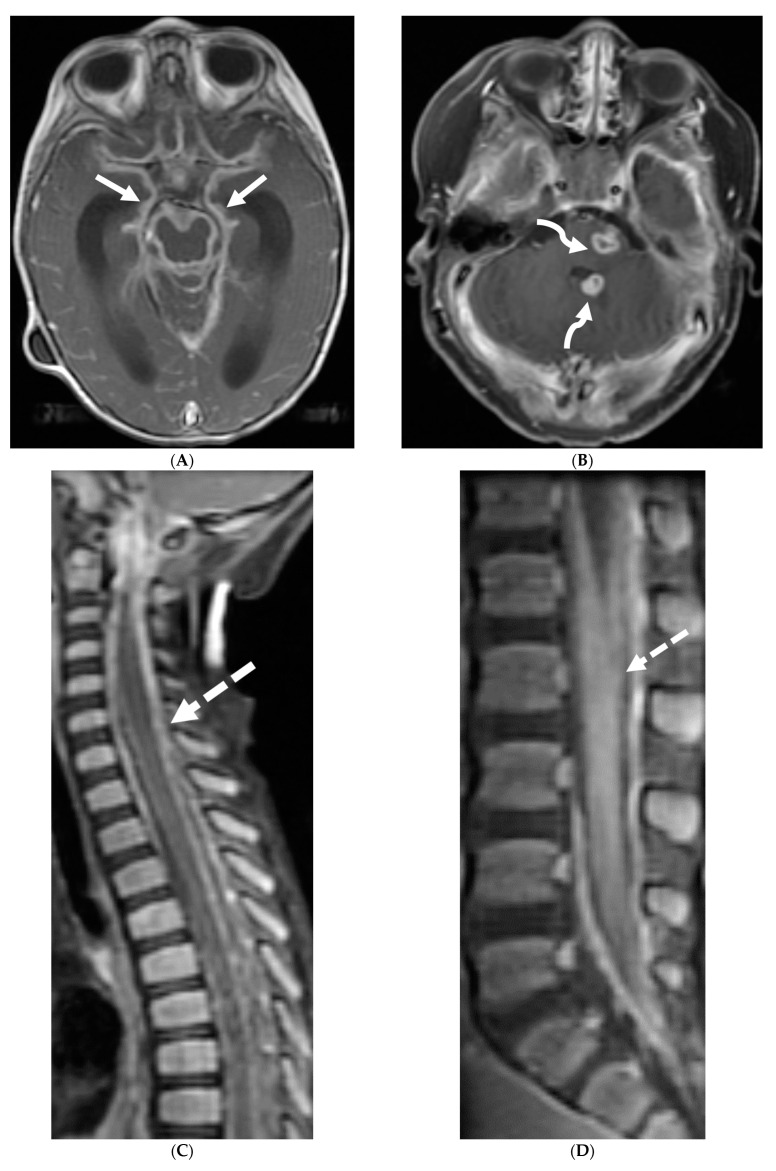
Axial T1 FS (**A**,**B**) and sagittal T1 fat sat (**C**,**D**). 2-year-old girl presented with emesis, fever and status epilepticus. Septic work up revealed tubercular meningitis. Extensive abnormal enhancement is seen in the meninges, prominent in the basilar cisterns (arrows). Ring-enhancing tuberculomas are seen in the cerebellum adjacent to the fourth ventricle and in the brainstem (curved arrows). Diffuse meningeal enhancement and thickening throughout the spinal canal as well as enhancement of the nerve roots is seen (dashed arrows).

TBM is characterized by LME, predominantly smooth and localized in the basal cisterns [76]. In severe cases, this can lead to the formation of basal exudates, visible as enhancing fluid in the basal cisterns [77]. The disease is often accompanied by parenchymal abnormalities, most commonly conglomerated ring-enhancing lesions with characteristic T2 hypointensity due to caseous content, although liquefaction may cause the core to become T2 hyperintense [78]. These ring-enhancing lesions can potentially coalesce to form abscesses. TBM may also present with infarcts in the thalami or basal ganglia due to basal vasculitis (Figure 13) [78]. The meningeal involvement typically occurs through hematogenous spread, and the condition is most prevalent in children and young adults presenting with altered mental status [79]. These imaging findings along with lymphocyte predominant CSF and significantly high protein can be diagnosed as tuberculosis since culture can take weeks.

### 4.3. Primary Brain Tumor Leptomeningeal Metastases (LM)

Common causes of leptomeningeal metastases from a pediatric intracranial primary tumor are medulloblastoma, embryonal tumor, ependymoma, germinoma, pineal tumor and atypical teratoid rhabdoid tumor. The primary theory suggests that tumor cells breach the pia mater and ependyma, gaining access to the cerebrospinal fluid (CSF). These cells then disseminate via the CSF, eventually settling along the spinal meninges [80]. The most common locations are thus, lower-thoracic and lumbar spine, located along the dorsal cord as CSF flow is from brain to the spine dorsally [81].

**Figure 14:**
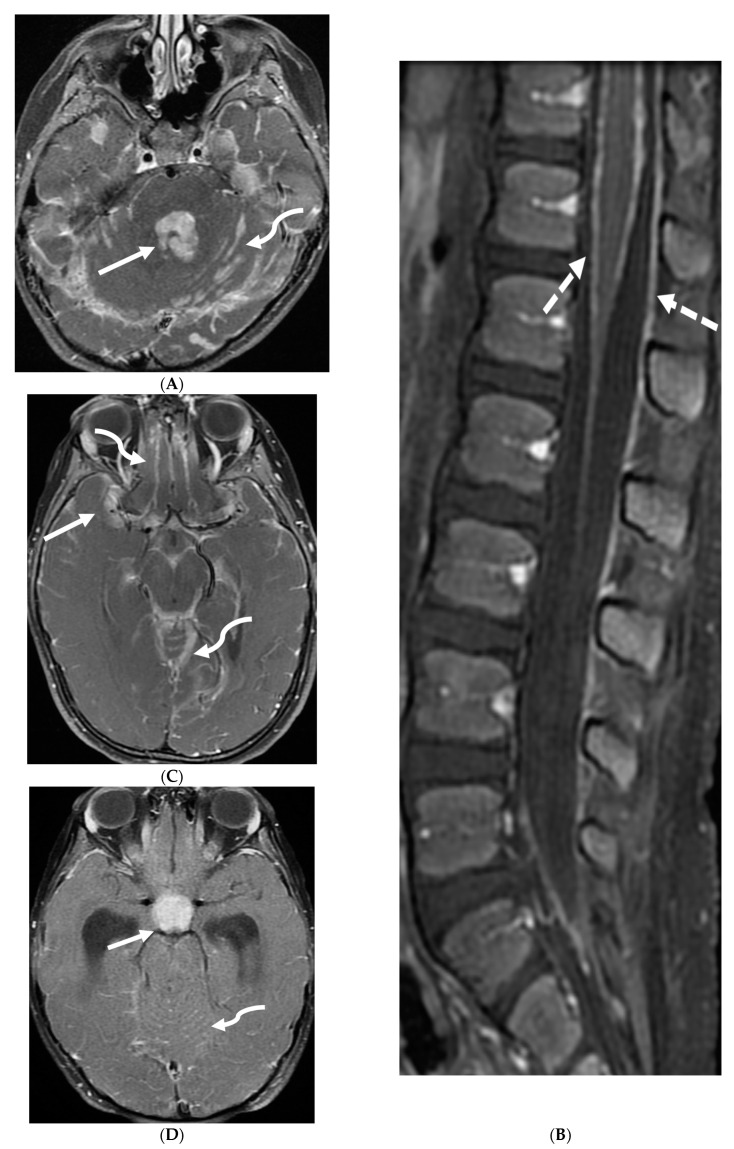
Post contrast axial T1 (**A**) and sagittal T1 (**B**): 3-year-old girl presented with headache, vomiting for 2 weeks and new right sided weakness. Fourth ventricular mass (arrow) with leptomeningeal metastasis (curved arrows). Dural and leptomeningeal metastasis (dashed arrows). Pathology: Anaplastic Medulloblastoma. Axial T1 post contrast (**C**): 4-year-old girl with headache and vomiting for 2 weeks. There is a partially enhancing mass in the right anterior temporal lobe (arrow) with extensive basal and leptomeningeal metastasis (curved arrow). Pathology: Atypical Teratoid Rhabdoid tumor (ATRT). Axial T1 post contrast (**D**): 10-year-old boy with vomiting and headaches: There are synchronous tumors in the suprasellar (arrow) and pineal region with hydrocephalus. Subtle LME is seen in the superior vermis (curved arrows). Pathology: Germinoma.

Currently, contrast-enhanced MRI and CSF cytology are the gold standards for detecting LM [82]. Key imaging findings include enhancing circumscribed nodule/s and/or irregular, thickened enhancement along the dorsal spine [81]. Pitfalls in detection of LM include vascular structures along the cord, seen as short segments of faint and thin enhancement. Veins are typically in midline, are tortuous and most prominently seen in high thoracic and conus medullaris regions (Figure 14). CSF flow artifacts can mimic LM on MRI, especially in the dorsal epidural space with a wide spinal canal. However, their epicenter in the subarachnoid space location can help distinguish them from true lesions [81,83]. MRI with 3D sequences offers superior sensitivity compared to CSF cytology, significantly reducing artifacts [80].

### 4.4. Systemic Meningeal Metastases (SMM)

Meningeal metastases from an extracranial primary tumor is a diagnosis with a grave prognosis, reducing the survival to less than 3 months if left untreated. Early and accurate diagnosis is essential for improving patient outcomes [84]. Leukemias and neuroblastoma are the most common primary malignancies in the pediatric population [85,86]. Leukemia is the only one which merits prophylactic treatment to avoid SMM.

**Figure 15:**
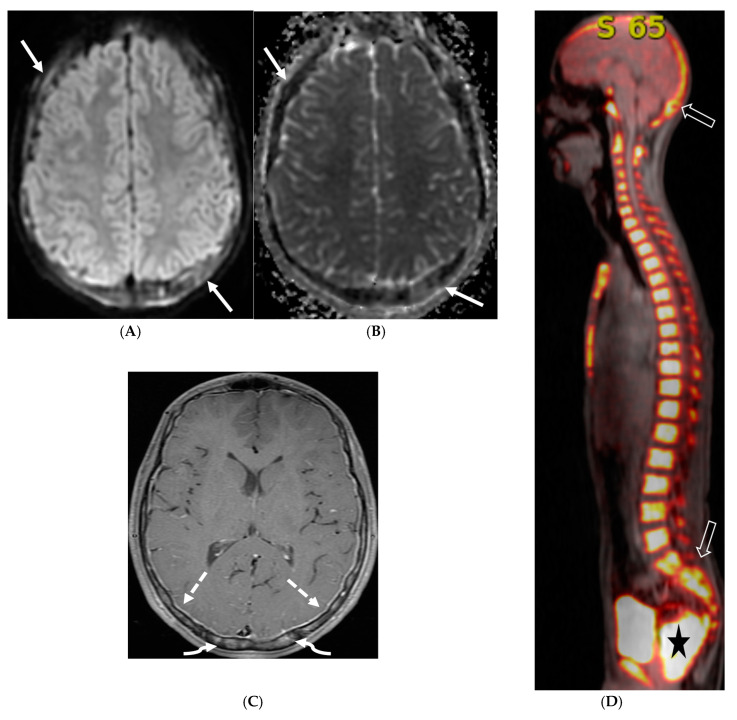
Axial DWI (**A**), axial ADC (**B**), axial T1 FS post contrast (**C**) and Fluorodeoxyglucose Positron Emission Tomography (FDG–PET) scan (**D**): 13-year-old male with bilateral leg pains, headache, fever and weight loss: Blood tests and CT scan were concerning for Burkitt’s lymphoma. There is heterogeneous calvarial bone marrow signal with restricted diffusion (arrows) and patchy enhancement (curved arrows). Diffuse thickening and enhancement of pachymeninges in the supratentorial compartment is noted (dashed arrows). Findings are most consistent with lymphomatous involvement. Multifocal diffuse/heterogeneous pattern of FDG uptake within the axial and appendicular skeleton and the calvarium (open arrows). Intense FDG avid uptake is seen in the presacral mass (star).

**Figure 16:**
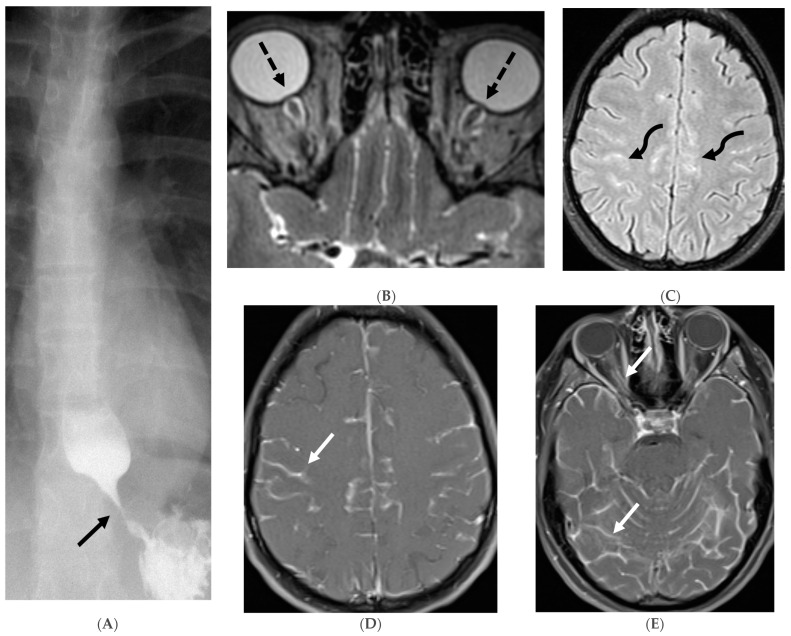
16-year-old female with 4 months history of globus sensation and recent botox injection of lower esophageal junction. Headache and vomiting for past week: Esophagogram (**A**), Axial T2 orbits (**B**), axial FLAIR (**C**) and post contrast T1 (**D**,**E**): Narrowing of the Gastroesophageal (GE) junction with beaked configuration and mild distention of the lower esophagus likely from early achalasia (black arrow). There is bilateral papilledema indicating raised ICP (dashed arrows) and sulcal hyperintensity (curved black arrows). Diffuse LME in the supra-and-infratentorial regions and along optic sheaths raising the concern for leptomeningeal carcinomatosis (white arrows). Pathology: Gastric adenocarcinoma metastasis.

**Figure 17:**
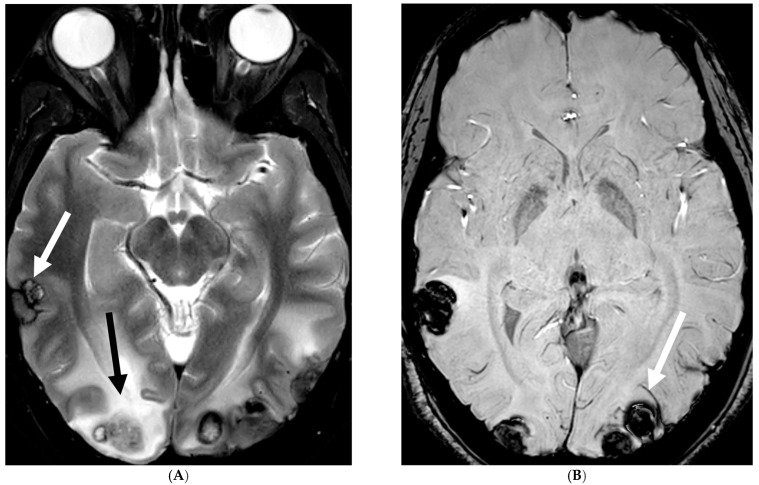

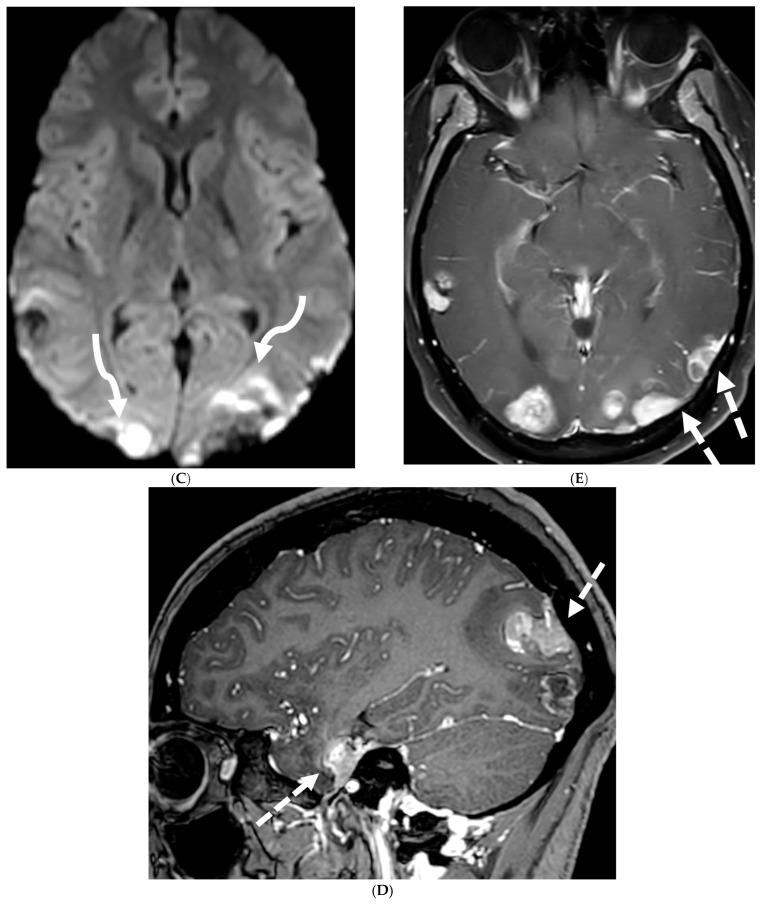
Axial T2 FS (**A**), axial Susceptibility weighted imaging (SWI) (**B**), axial DWI (**C**), post contrast sagittal and axial T1 post contrast (**D**,**E**): 19-year-old female with history of stage IV neuroblastoma, left paraspinal primary ganglioneuroblastoma, treated with chemotherapy, radiation and bone marrow transplant presents with headache. There are extensive hemorrhagic leptomeningeal masses, both supra and infratentorial region (arrows). The lesions also demonstrate restricted diffusion which could be secondary to internal hemorrhage or high cellularity of the tumor(curved arrows). The larger masses invade the cortex of both cerebral hemispheres, with surrounding vasogenic edema (black arrow). Avid enhancement of the lesions is seen along with overlying dura (dashed arrows). Features are in keeping with extensive leptomeningeal metastatic neuroblastoma.

The typical MRI presentation of SMM involves serpentine, nodular, or plaque-like enhancement in sulcal spaces, basal cisterns and along the cauda equina nerve roots [87,88]. Notably, SMM enhancement on post contrast [PC]-T1 images is superior to the PC-FLAIR in contrast to that seen in infectious meningitis [84]. However, in case of a non-enhancing primary tumor, FLAIR and DWI are important sequences to look for SMM. Hydrocephalus and subependymal deposits are other common features found in kids with SMM (Figure 15, Figure 16 and Figure 17). Positive cytology on CSF analysis, especially with leukemia, is important [89].

### 4.5. Moya Moya

Moya moya disease (MMD) is a complex neurovascular condition characterized by progressive narrowing of the internal carotid terminus and/or the proximal anterior or middle cerebral arteries. Due to chronicity, arterial collateral vessels develop to bypass the narrowing. These collaterals can be basal, leptomeningeal or transdural, arising from perforating arteries, typically coursing through the meninges [90]. These collaterals are often small, weak, and prone to bleed or clot.

MRI, the standard noninvasive imaging modality, shows vascular narrowing on T2-weighted images and collateral vessels [91,92]. FLAIR sequence helps in detecting subtle areas of gliosis or chronic white matter ischemia. While DWI remains the optimal sequence for detecting acute ischemia, SWI helps in detecting acute or chronic microbleeds in addition to prominent deep medullary veins in areas with impaired blood flow (depicted as the “brush sign”). MR angiography defines the disease extent.

**Figure 18:**
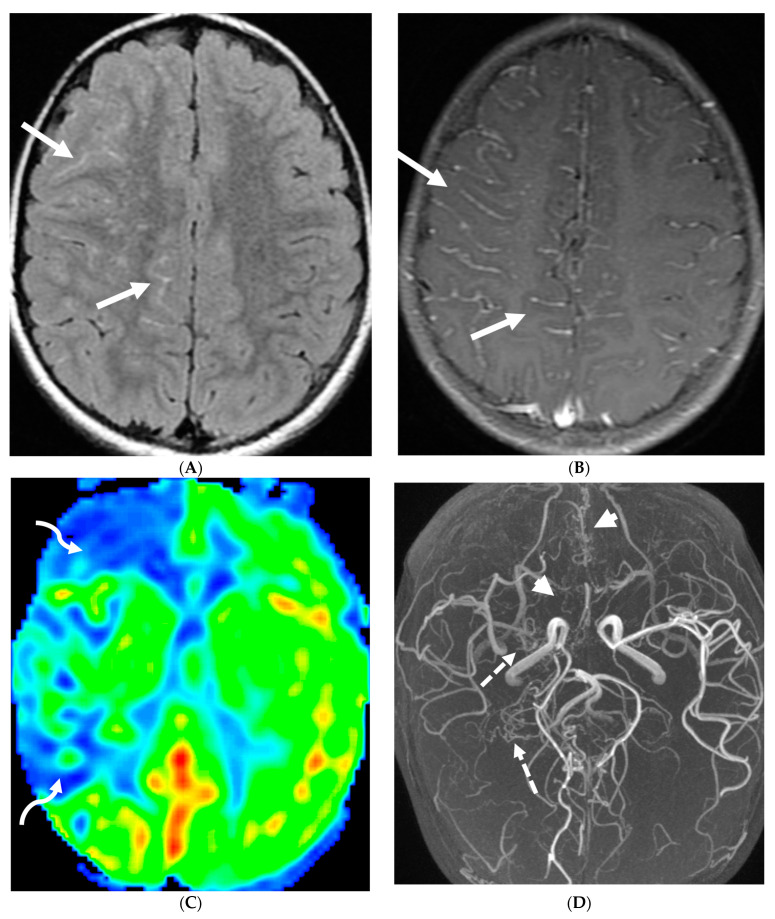
Axial FLAIR (**A**), MRA (**B**), axial ASL perfusion (**C**) and axial T1 post contrast (**D**): 7-year-old girl with Down’s syndrome: Abnormal FLAIR hyperintense signal with LME along the right cerebral convexity sulci, predominantly in the frontoparietal region representing “ivy sign” (arrows). Asymmetric decreased perfusion in the right frontal and temporal regions (curved arrows). The M1 segment of right MCA is not visualized with extensive moyamoya vessels (dashed arrows). The M2 and M3 branches of right MCA are asymmetrically attenuated. Bilateral A1 segments are not identified with extensive collateralization and diminutive caliber of A2 and A3 segments (arrow heads).

In children with moya moya, the LME is termed the “ivy sign” as the appearance resembles creeping ivy (Figure 18) [93]. LME arises due to two key factors in MMD, namely neovascularization and retrograde flow from congested pial vessels [94].

Although LME is a supportive feature in the diagnosis of moya moya, LME is a marker of collateral vessel status and less LME is a marker of severe clinical symptoms and poor postoperative outcomes [95]. In addition, degree of reduction of LME after surgery has been proposed to be a marker of effective surgery [96].

## 5. Prominenet Parenchymal Features

### 5.1. Viral Meningitis

Several viruses, such as enterovirus, herpes simplex virus (HSV)-1&2, mumps, varicella, and arbovirus, can infect children, out of which enterovirus is the most common. These organisms have variable LME, ranging from none to diffuse sulcal LME, best demonstrated on post contrast FLAIR over T1 images [97]. HSV is associated with poor prognosis due to associated parenchymal involvement. HSV-1 commonly causes oral herpes in contrast to HSV-2 which typically causes genital herpes in adults. An active/remote HSV 2 infection in the mother increases the risk of neonatal transmission if delivered vaginally.

**Figure 19:**
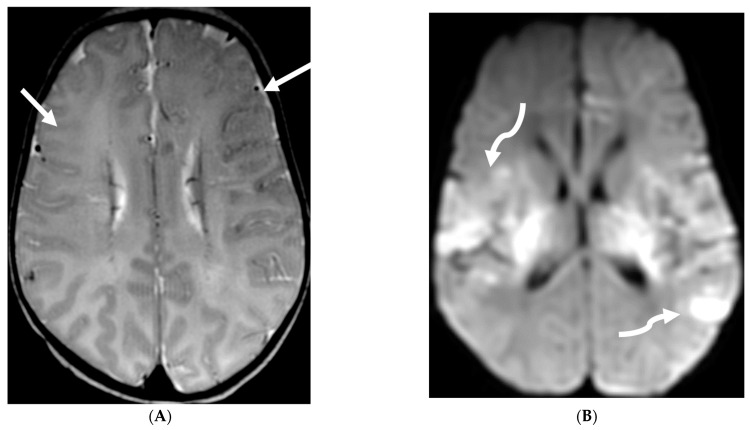

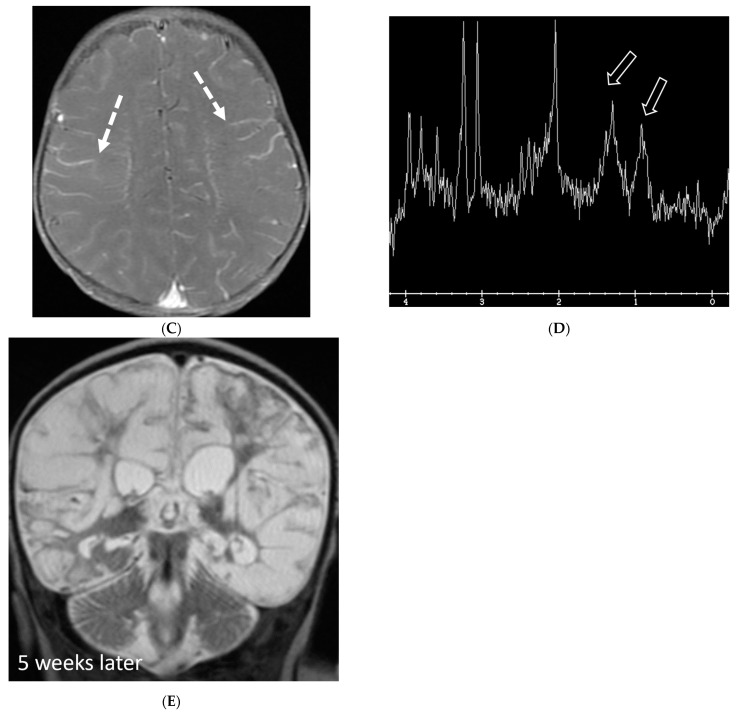
17-day-old girl with seizures. Axial T2 (**A**), axial DWI (**B**), axial T1 post contrast (**C**), short TE spectroscopy (**D**) and coronal T2 (**E**): There is loss of gray white matter differentiation indicating edema in bilateral frontal lobes (arrows). Extensive ischemic changes involving bilateral frontal, bilateral parietal lobes, bilateral perisylvian regions, bilateral thalami (curved arrows). Extensive LME is identified in the effected regions (dashed arrows). Abnormal elevation of lipid/lactate in both basal ganglia and white matter (open arrows). The above constellation of features are concerning for meningitis/cerebritis. Follow up MRI 5 weeks later demonstrates evolution of extensive ischemic changes into extensive cystic encephalomalacia and gliosis in the supratentorial brain, with ex vacuo enlargement of the ventricular system. CSF analysis: HSV-2.

HSV 2 typically causes diffuse cortical involvement with diffusion restriction, loss of gray white matter differentiation and basal ganglia involvement in early stages [98]. HSV 1 typically occurs in older children and adolescents and leads to asymmetric temporal lobe involvement with relative sparing of the basal ganglia (Figure 19).

### 5.2. Fungal Meningitis

Fungal infections of the central nervous system (CNS) can be broadly categorized into two forms based on the causative organism’s size and pathogenesis. Yeast infections (e.g., Cryptococcus, Candida) are smaller and disseminate hematogenously, resulting in parenchymal granulomas, abscesses, and diffuse leptomeningitis. Mold infections (e.g., Aspergillus, Mucorales) are larger fungi that are restricted from entering the meningeal microcirculation, leading to more focal disease manifestations such as cerebritis, abscess formation, vasculitis, infarct, and mycotic aneurysm [99]. Candida is the most common fungal organism affecting children, typically in preterm and/or low birth weight neonates [100]. The routes of CNS invasion by fungal pathogens include hematogenous dissemination from a distant source (commonly lung), direct inoculation following trauma or neurosurgical procedures, and local extension from adjacent structures like the paranasal sinuses, orbit, or spine [101].

**Figure 20:**
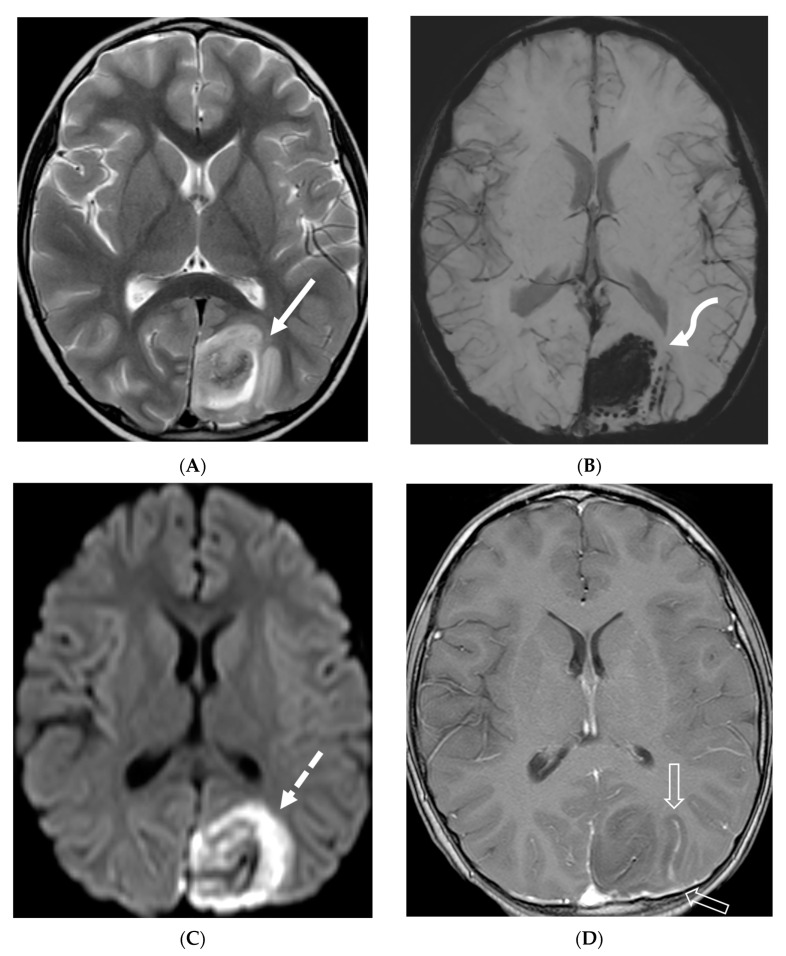
Axial T2 (**A**), axial DWI (**B**), axial SWI (**C**) and axial T1 post contrast (**D**): 4-year-old girl with acute lymphoblastic leukemia, pancytopenia and fever. Treatment started one week before with asparaginase. There is prominently T2 hyperintensity and swelling of the gyri involving the medial aspect of the left parietal occipital cortex (arrow). Multiple small foci of T2 hypointensities are identified within the involved region with corresponding blooming on the susceptibility indicating hemorrhage (curved arrow) and peripheral rim of true restricted diffusion (dashed arrow). Postcontrast images show pachymeningeal and LME in the involved region (open arrows). Features are concerning for fungal infection. Pathology revealed Rhizomucor pusillis (thermophilic fungus).

MRI findings in fungal meningitis include LME, which can be smooth or thick, nodular and irregular, and commonly involve the cortical sulci [102]. While a smooth, linear enhancement pattern is common in viral and bacterial meningitis, it can also be seen in immunocompetent patients with fungal meningitis [102]. Inflammatory exudates containing cell debris, fibrin, and hemorrhage can deposit in the subarachnoid space, leading to arachnoiditis. Protein accumulation in the subarachnoid space shortens T1 relaxation time and results in increased signal intensity on FLAIR sequences [102]. Meningeal adhesions can obstruct arachnoid granulations, leading to impaired cerebrospinal fluid (CSF) drainage and secondary hydrocephalus. Fungal brain abscesses typically demonstrate a central T1 hypointense and T2 hyperintense core, surrounded by a T1 iso-to-hyperintense and T2 hypointense enhancing peripheral rim (Figure 20) [103,104].

### 5.3. Anti-Myelin Oligodendrocyte Glycoprotein (MOG) Demyelination

Anti-MOG antibody associated demyelination (MOGAD) frequently presents as Acute Disseminated Encephalomyelitis (ADEM) in children and opticospinal involvement in young adults [105]. Bilateral but asymmetric T2 hyperintense lesions occur in thalamus, pons and cerebellar peduncles are common in children [106]. Optic nerve involvement typically presents as a long segment with anterior predominance, in contrast to the posterior predominance seen in Neuromyelitis Optica Spectrum Disorders (NMOSD) and the short segment involvement characteristic of Multiple Sclerosis (MS) [106].

**Figure 21:**
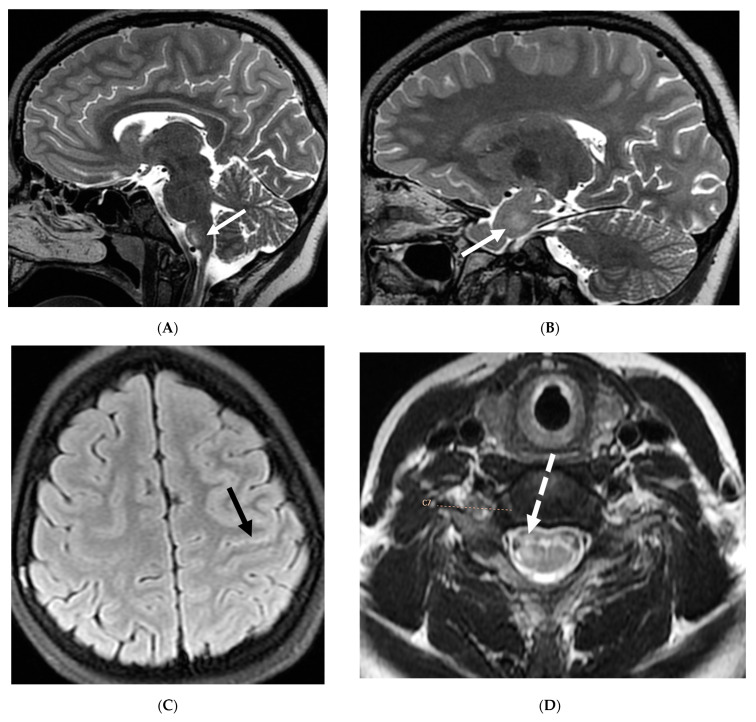

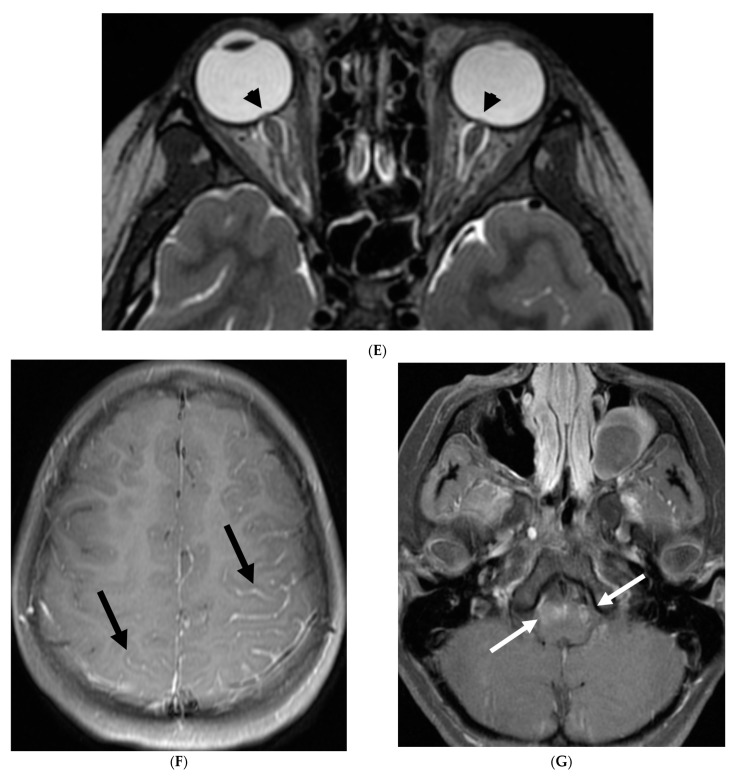

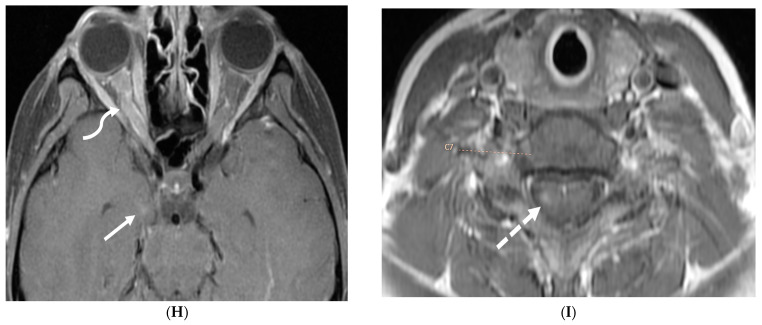
Sagittal T2 (**A**,**B**), axial FLAIR (**C**), axial T2 cervical spine (**D**) at the level of C7 vertebral body and Axial T2 orbits (**E**): 12-year-old girl presented with right focal motor seizure and left temporal lobe slowing on electroencephalogram (EEG). Right eye vision loss and irritability. Ill-defined areas of signal abnormalities are identified within the RIGHT mesial temporal lobe and bilateral medulla (white arrows). FLAIR hyperintensity is identified on the left central sulcus (black arrow). Small focus of signal abnormality is seen on the right side of the cord at the 7th cervical vertebra (C7) (dashed arrow). There is also bilateral papilledema (arrowheads). Post contrast axial T1 (**F**,**G**), axial T1 orbits (**H**) and axial T1 cervical spine at C7 (**I**): Asymmetric LME (black arrows) predominantly involving the left cerebral hemisphere, with minimal right parietal involvement is seen. Ill-defined enhancement in the right mesial temporal lobe, and right greater than left medulla (white arrows) corresponds to the signal abnormality. There is right greater than left, optic nerve enhancement (curved arrow). Single small enhancing lesion in the spinal cord on the right at the level of C7 corresponds to the signal abnormality (dashed arrow). Features favor a demyelinating process. MOG antibodies were positive at 1:20 in keeping with Myelin oligodendrocyte glycoprotein (MOG) antibody disease (MOGAD).

LME has been shown to present early in the disease course and is much more common in children (33%) compared to adults (8%) [107]. Gadde et al. found that 8% of pediatric MOG antibody-associated demyelination cases had only LME without any other central nervous system manifestation. LME when present can be particularly helpful in differentiating from NMOSD [106]. Furthermore, Valencia-Sanchez et al. reported a significant association between LME and cerebral cortical encephalitis in MOG antibody-associated disease. This finding suggests that LME may be an important marker for cortical involvement and potentially more severe disease (Figure 21) [108].

### 5.4. Granulomatosis Polyarteritis (GPA)

GPA is an autoimmune necrotizing granulomatous inflammation associated with anti-neutrophil cytoplasmic antibody (ANCA) vasculitis. This multisystem disorder predominantly affects small vessels, causing endothelial injury and tissue damage in the upper and lower respiratory tract and renal system [109,110]. Neurologic involvement occurs in 20–50% of GPA patients, mediated by three main mechanisms: vasculitis of cerebral vessels, granuloma formation due to contiguous involvement from adjacent paranasal and orbital lesions, and remote granulomatous lesions in brain parenchyma or meninges [111,112]. Patients typically present with headache, altered mental status, and transient ischemic attacks. Pituitary gland involvement can manifest as hyperprolactinemia or diabetes insipidus [111].

**Figure 22:**
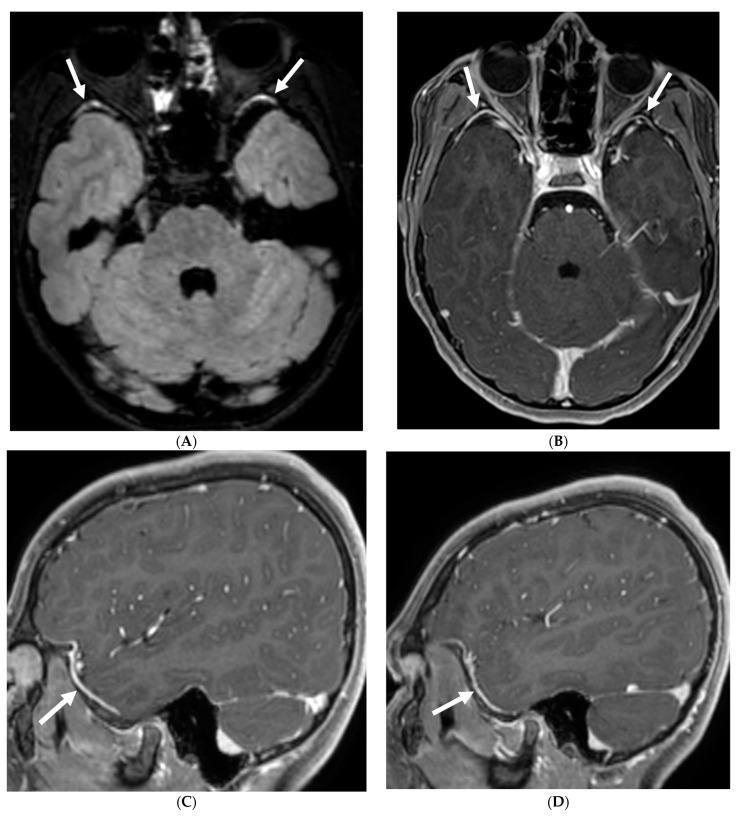
Post contrast axial FLAIR (**A**), axial T1 FS (**B**), sagittal T1 Right (**C**) and Left (**D**): 10-year-old girl with elevated ANCA, headache and mild LUE weakness. There is bilateral anterior temporal smooth dural enhancement (white arrows). Diagnosis: Antineutrophilic cytoplasmic antibody (ANCA) associated vasculitis, likely granulomatosis with polyangiitis.

Imaging findings in GPA include chronic hypertrophic pachymeningitis (most common) representing granulomatous involvement. This can be diffuse or focal, with the latter showing dural thickening and enhancement adjacent to a sinus or orbit [113]. Tentorium involvement is common, presenting as the ‘Eiffel by night’ sign [114]. Cerebral vasculitis appears as multiple T2 hyperintense white matter lesions potentially showing diffusion restriction and patchy enhancement. Cerebral atrophy may be observed, attributed to steroid treatment and/or vasculitis. Pituitary involvement can range from normal to enlarged gland size, with thickened stalk and peripheral enhancement. Cranial nerve involvement, particularly of the olfactory and optic nerves, is common due to mass effect from adjacent lesions or hypertrophic pachymeningitis (Figure 22) [112].

### 5.5. NMDA Encephalitis

Anti-N-methyl-D-aspartate receptor (NMDA) encephalitis is a subtype of limbic encephalitis, the other subtype being paraneoplastic [115]. This autoimmune response to NMDA receptors involved in excitatory neurotransmission results in a constellation of neuropsychiatric and neurological symptoms. It predominantly affects young females and children without an underlying malignancy, although in a subset of cases, particularly in young women, an ovarian teratoma may be associated [116].

The clinical presentation of Anti-NMDA encephalitis often begins with a prodromal phase resembling a viral illness, followed by the evolution of characteristic symptoms over days to weeks. These may include psychiatric manifestations; temporal lobe dysfunction manifesting as memory impairment and seizures; and severe neurological deficits such as autonomic instability and movement disorders (dystonia/dyskinesia) [117,118].

**Figure 23:**
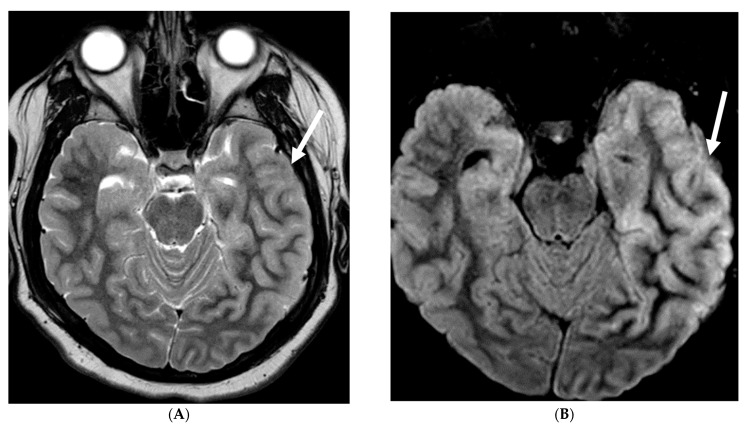

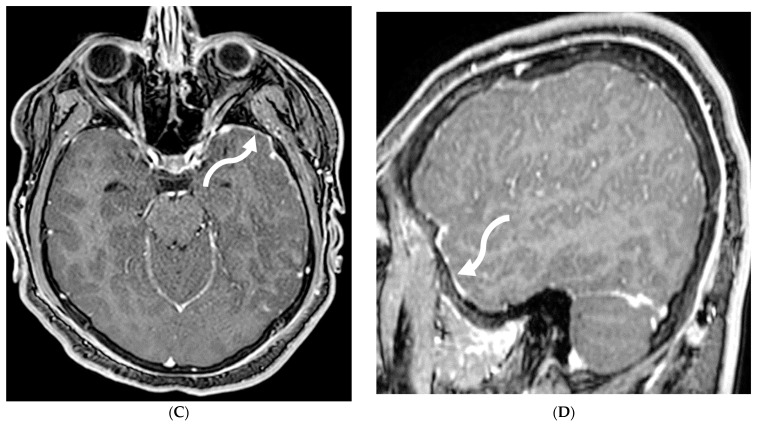
Axial T2 (**A**), post contrast FLAIR (**B**), axial and sagittal (left) T1 (**C**,**D**). 16-year-old male with 3 weeks of headache, photophobia and vomiting. There is asymmetric left cerebral swelling with cortical T2 hyperintensity (white arrows) and anterolateral left temporal LME (curved arrows). Diagnosis: NMDA receptor encephalitis (initially thought to be HSV).

MRI findings in Anti-NMDA encephalitis are frequently nonspecific or absent [119]. However, potential imaging abnormalities may include transient cortical signal enhancement involving the hippocampi, cerebellum, cerebral cortex, insular regions, periventricular white matter, basal ganglia, or brainstem [120]. LME, reflecting meningeal inflammation, may also be observed in conjunction with parenchymal changes (Figure 23) [121]. Notably, the absence of restricted diffusion and hemorrhage on MRI can aid in differentiating Anti-NMDA encephalitis from other etiologies, such as viral encephalitis [120]. It is crucial to recognize that a normal MRI does not exclude the diagnosis of Anti-NMDA encephalitis.

### 5.6. Posterior Reversible Encephalopathy Syndrome (PRES)

PRES, is a reversible encephalopathy characterized by vasogenic edema, predominantly in the posterior cerebral white matter [122]. The pathophysiology of PRES is likely an autoregulatory dysfunction and/or vasoconstriction of cerebral arteries [123]. Clinical presentation includes altered consciousness, seizures, headache, and visual disturbance, often developing abruptly and resolving within weeks with appropriate management. The most common predisposing factor is hypertension, particularly with abrupt or intermittent increase in blood pressure [123]. Additionally, nephrotic syndrome, particularly during relapses, is a risk factor due to calcineurin inhibitor use, hypertension, and increased vascular permeability [123].

**Figure 24:**
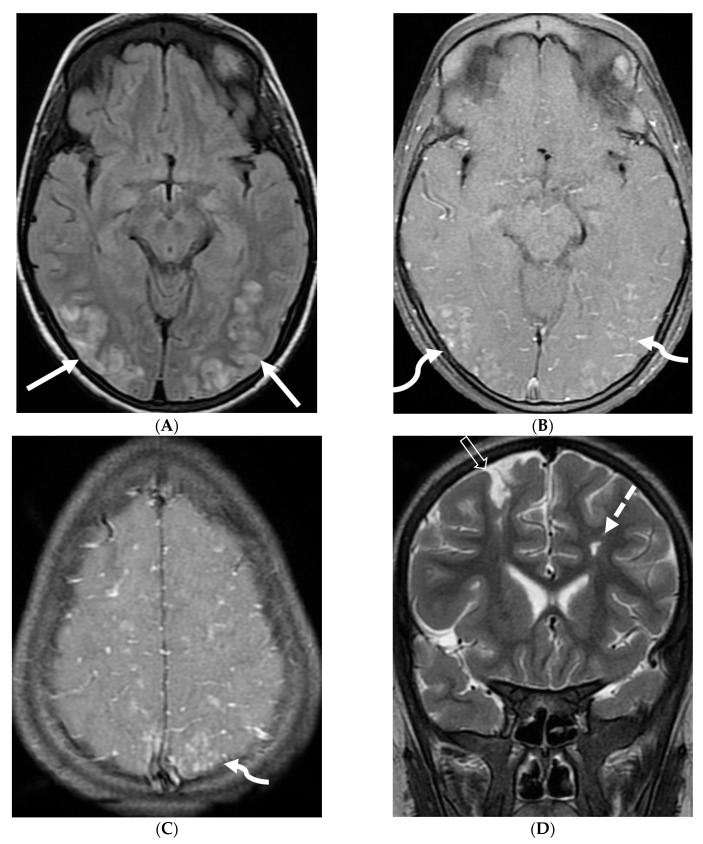
Axial FLAIR (**A**), post contrast axial T1 (**B**,**C**) and coronal T2 (**D**): 6 year 9-month-old male with sickle cell disease presented with altered mental status, seizure and hypertension. Multiple areas of T2/FLAIR signal hyperintensities are seen in a relatively symmetric distribution involving the bilateral occipital, posterior parietal, high frontal and posterior temporal lobes (arrows). Multiple areas of LME are demonstrated in the involved regions (curved arrows). These findings are characteristic of posterior reversible encephalopathy syndrome (PRES). Areas of encephalomalacia and gliosis involving the deep white matter of bilateral frontal lobes (dashed arrow) and a small area of old cortical infarct involving the right frontal lobe (open arrow), secondary to small vessel disease in a patient with sickle cell disease.

MRI is the primary imaging modality for detecting PRES [13,16]. T2-weighted and FLAIR images show hyperintense foci in the posterior parietal and occipital lobes, but also frequently involving other regions, including the frontal and inferior temporal lobes and cerebellum [124]. Few tiny to small foci of diffusion restriction may also occur. As per Agarwal et al., leptomeningeal FLAIR signal was seen in about one third of the patients with post contrast enhancement (leptomeningeal +/− cortical) in about 25% of the total population. Majority of these were isolated and had no vasogenic edema [122]. In addition, increased gadolinium dose and delayed imaging increase the incidence of LME (Figure 24) [125].

### 5.7. Pial Angiomatosis

Pial angiomatosis is the hallmark of Sturge-Weber syndrome (SWS), a neurocutaneous disorder characterized by facial port-wine birthmark, ocular abnormality (choroidal angiomas), and leptomeningeal vascular malformation. The pathogenesis involves abnormal persistence and proliferation of embryonic vascular plexuses within the leptomeninges, resulting in tangled angiomatous growths [126]. There is lack of proper venous drainage, leading to rerouting of blood flow through the compensatorily dilated deep medullary veins resulting in venous hypertension and ischemic injury to the underlying cerebral cortex.

MRI with contrast is the preferred modality for evaluation of pial angiomatosis. Early disease stages may show increased cerebral blood flow/volume, characteristic accelerated myelination, LME, and restricted diffusion indicating acute ischemia [127]. LME is thought to be secondary to venous stasis, decreased blood brain barrier or transiently following seizure [128]. Late stage findings include subcortical calcifications, cortical atrophy, prominent deep medullary veins, and ipsilateral choroid plexus enlargement [126].

**Figure 25:**
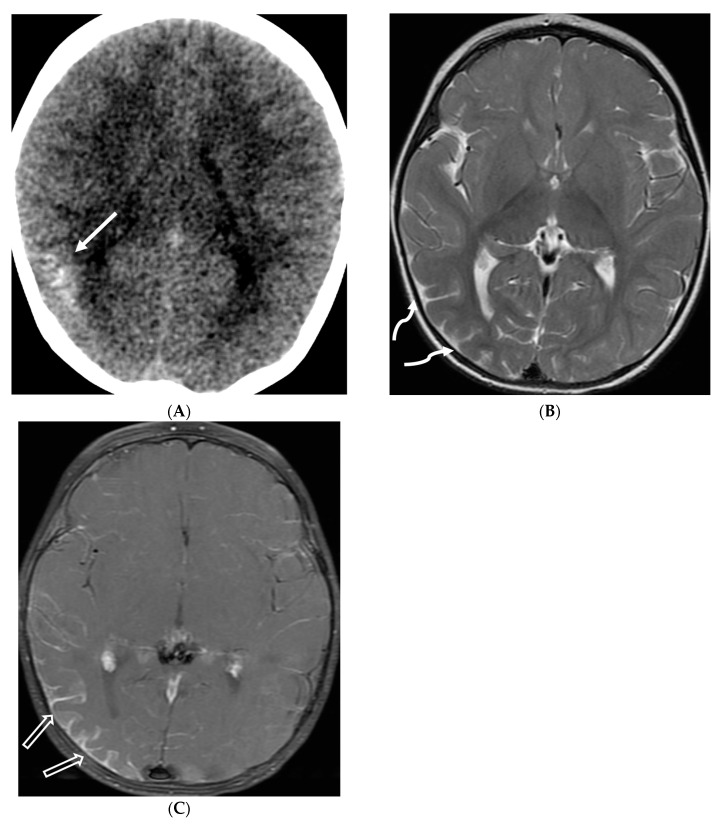
Axial CT (**A**), Axial T2 (**B**) and Axial T1 post contrast (**C**): 12-month-old girl presented with focal left sided seizures. There is curvilinear calcification in the right temporal lobe with cortical volume loss (arrow). There is mild parenchymal volume loss and dysmyelination in the right temporal, occipital, and parietal lobes (curved arrows). Thick pial enhancement is seen in the corresponding areas (open arrows). Findings in keeping with pial angiomatosis in the right temporal, occipital, and parietal lobes. Note: Patient does not have port wine stain to support the diagnosis of Sturge-Weber syndrome.

Characteristic findings on susceptibility-weighted imaging (SWI) are serpentine calcifications along the cerebral gyri [128]. While pial enhancement on postcontrast MRI is the gold standard, some patients with suspected SWS may lack this finding initially, with pial angiomatosis only becoming evident on follow-up imaging (Figure 25).

### 5.8. Langerhans Cell Histiocytosis (LCH)

LCH is an uncommon, often systemic pediatric disorder [129,130]. Clinical course can vary from spontaneous resolution, chronic recurrence to rapid and fatal progression [131]. LCH most frequently affects the bone (80%), the skin (33%), and the pituitary gland (25%) [132]. CNS involvement is seen in 25–50% of cases of LCH [133]. Clinical symptoms depend on the site of CNS involvement. Diabetes insipidus is the most common manifestation followed by growth hormone deficiency [134].

The imaging manifestations of CNS LCH can be categorized into four groups [135]. Cranio-facial osteolytic lesions having typical beveled margins with or without a soft tissue component is most prevalent. Hypothalamic-pituitary region is the most frequently involved intracranial structure correlating with anterior pituitary hormone deficiency and diabetes insipidus [136]. There is thickening of the pituitary stalk due to infiltration by LCH granulomas, which may progress to space occupying pituitary or hypothalamus mass. The loss of Anti Diuretic Hormone granules corresponds to the loss of T1 hyperintense posterior pituitary bright spot. Meningeal lesions occur in less than one third of children with LCH, often adjacent to soft tissue or osseous lesions with T1 intermediate and T2 hyperintensity signal intensity and homogeneous enhancement [135].

**Figure 26:**
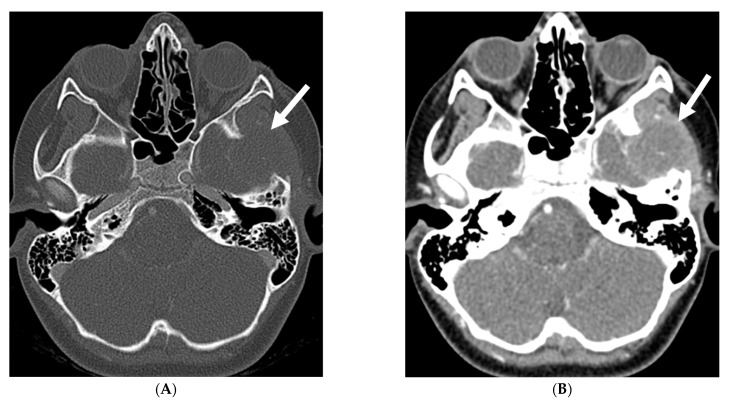

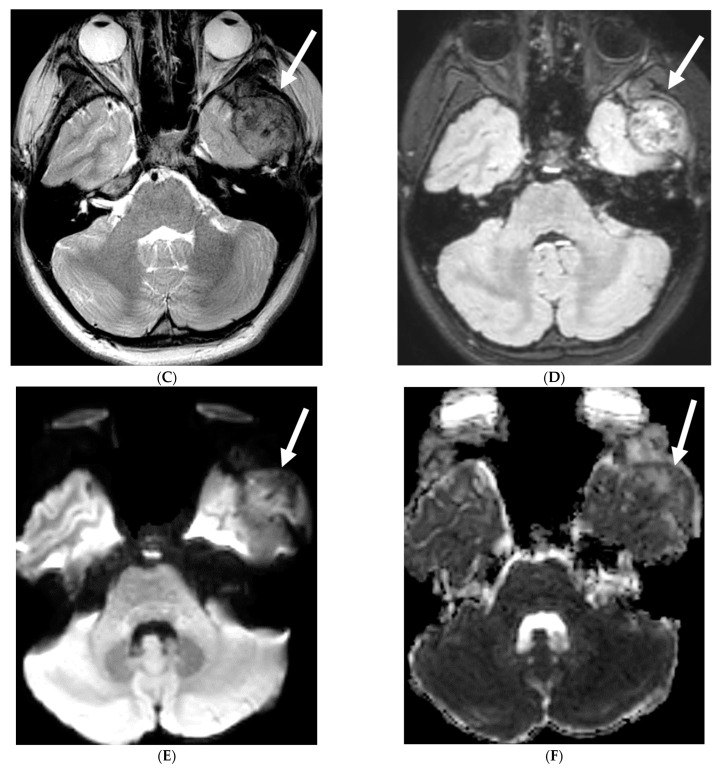

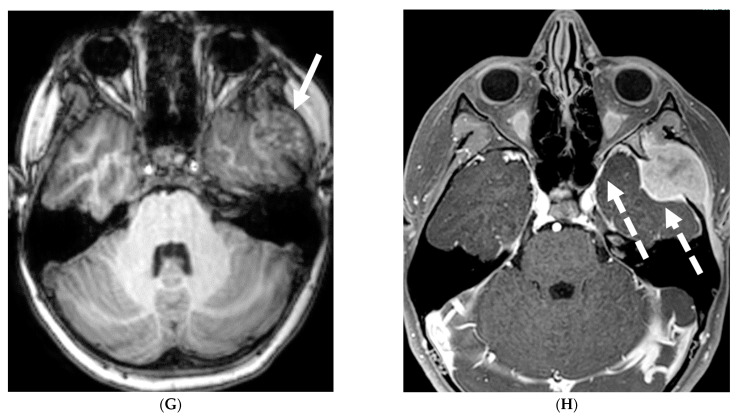
Axial CT bone (**A**) and brain (**B**) windows, Axial T2 (**C**), Axial FLAIR (**D**), Trace DWI (**E**), ADC (**F**), Axial T1 pre (**G**) and post contrast (**H**): 9-year-old boy with palpable left cheek mass. There is an ovoid destructive mass (white arrows) in the greater sphenoid wing bulging into the middle cranial fossa with heterogeneous slight restricted diffusivity. The lesion avidly enhances with dural thickening and enhancement (dashed arrows) extending toward Meckel cave and the cavernous sinus. Pathology: LCH.

Circumventricular region which includes pineal gland, choroid plexus and ependyma and are located outside blood-brain-barrier. The concurrent involvement of pituitary and pineal gland can be due to functional interaction and direct infiltration by the disease process [137]. Leukoencephalopathy pattern involving the cerebellar white matter (most common), pons, and/or periventricular white matter can be seen as symmetric patchy T2 hyperintense and T1 hypointense lesions [135,136]. Cerebellar atrophy can also be seen (Figure 26) [138].

## 6. Conclusions

Pediatric meningeal diseases exhibit overlapping imaging features that pose diagnostic challenges. An imaging-based classification, emphasizing parenchymal and associated findings, can aid in systemic evaluation. Integrating these radiological patterns with clinical and laboratory data helps improve diagnostic accuracy. This approach is crucial for guiding patient management, particularly in acute settings where imaging often precedes definitive diagnostic tests.

## Figures and Tables

**Table 1:** Proposed Classification to differentiate meningeal diseases in children based on imaging features.

	Prominent Meningeal Features	Variable	Prominent Parenchymal Features
Infectious	Viral [Except HSV]	Group B Streptococci	HSV
	Algae (Prototheca)	Tuberculosis	Fungal
Autoimmune	Neurosarcoid		Anti-MOG Demyelination
	Guillian Barre Syndrome		ANCA vasculitis
	Idiopathic Hypertrophic Pachymeningitis		NMDA Encephalitis
Neoplastic	Meningioma	Drop Metastasis (Primary CNS tumors)	
	Glioneuronal tumor	Systemic Metastasis	
	Meningeal Rhabdomyosarcoma		
Vascular		Moya Moya disease	PRES
			Pial Angiomatosis
Other	Intracranial hypotension		LCH
	ALK positive Histiocytosis		

HSV—Herpes Simplex Virus 2; Anti-MOG—Anti-Myelin Oligodendrocyte Glycoprotein; ANCA—Antineutrophil Cytoplasmic antibody; NMDA—N-methyl-D-aspartate; PRES—Posterior Reversible Encephalopathy Syndorme; ALK—Anaplastic Lymphoma Kinase; LCH—Langerhans Cell histiocytosis; CNS—Central Nervous System.
